# Physical and Biological Controls on the Carbonate Chemistry of Coral Reef Waters: Effects of Metabolism, Wave Forcing, Sea Level, and Geomorphology

**DOI:** 10.1371/journal.pone.0053303

**Published:** 2013-01-09

**Authors:** James L. Falter, Ryan J. Lowe, Zhenlin Zhang, Malcolm McCulloch

**Affiliations:** 1 School of Earth and Environment, University of Western Australia, Perth, Western Australia, Australia; 2 ARC Centre of Excellence for Coral Reef Studies, University of Western Australia, Perth, Western Australia, Australia; 3 The UWA Oceans Institute, University of Western Australia, Perth, Western Australia, Australia; University of Vigo, Spain

## Abstract

We present a three-dimensional hydrodynamic-biogeochemical model of a wave-driven coral-reef lagoon system using the circulation model ROMS (Regional Ocean Modeling System) coupled with the wave transformation model SWAN (Simulating WAves Nearshore). Simulations were used to explore the sensitivity of water column carbonate chemistry across the reef system to variations in benthic reef metabolism, wave forcing, sea level, and system geomorphology. Our results show that changes in reef-water carbonate chemistry depend primarily on the ratio of benthic metabolism to the square root of the onshore wave energy flux as well as on the length and depth of the reef flat; however, they are only weakly dependent on channel geometry and the total frictional resistance of the reef system. Diurnal variations in *p*CO_2_, pH, and aragonite saturation state (Ω_ar_) are primarily dependent on changes in net production and are relatively insensitive to changes in net calcification; however, net changes in *p*CO_2_, pH, and Ω_ar_ are more strongly influenced by net calcification when averaged over 24 hours. We also demonstrate that a relatively simple one-dimensional analytical model can provide a good description of the functional dependence of reef-water carbonate chemistry on benthic metabolism, wave forcing, sea level, reef flat morphology, and total system frictional resistance. Importantly, our results indicate that any long-term (weeks to months) net offsets in reef-water *p*CO_2_ relative to offshore values should be modest for reef systems with narrow and/or deep lagoons. Thus, the long-term evolution of water column *p*CO_2_ in many reef environments remains intimately connected to the regional-scale oceanography of offshore waters and hence directly influenced by rapid anthropogenically driven increases in *p*CO_2_.

## Introduction

Rapidly rising levels of atmospheric CO_2_ are expected to continue decreasing seawater pH and carbonate mineral saturation states across the world's oceans through a process commonly referred to as ‘ocean acidification’ [1]–[3]. Such trends have caused particular concern for the sustainability of coral reefs; ecosystems whose function and structure are ultimately dependent upon the biogenic precipitation of calcium carbonate [4]–[6]. Consequently, the sensitivity of calcification to changing carbonate chemistry has received considerable attention over the past decade. Much of this work has been carried out through manipulative experiments of live communities using aquaria or mesocosms where selected input variables can be carefully controlled [7]–[10]. The results of these studies have, however, demonstrated a wide range of variation depending not only on the calcifying species, but also on the experimental design [8], [11]. A recent process-based synthesis [12] suggests that the impact of ocean acidification will be bi-modal: i.e., healthy calcifiers capable of pH up-regulation at the site of calcification (e.g., coral) demonstrate a high degree of resilience to changes in ambient carbonate chemistry whereas those calcifiers lacking the ability to up-regulate pH (e.g., forams) appear far more sensitive to changes in ambient *p*CO_2_. Thus, predicting the future response of reef calcifiers to changing atmospheric *p*CO_2_ is still uncertain. Furthermore, there are additional interactive effects of increased *p*CO_2_ on the ecology of reef biota that extend beyond rates of skeletal growth, yet these still remain poorly understood [13]–[16].

It has been well-known for many decades that shallow reef communities can alter the chemistry of their environment through their own metabolic activity [17]–[19]. For example, Smith 1973 [20] first demonstrated that the *p*CO_2_ of reef waters are generally not in equilibrium with the atmosphere on short timescales, a direct outcome of community metabolism driving diurnal variations in water column carbonate chemistry (see Fig. 2 and Fig. 4 from that paper). Numerous subsequent studies have shown that the biological activity of reef communities can drive variations in *p*CO_2_ that range from levels below a pre-industrial atmosphere (<285 µatm) to greater than predicted for the earth's atmosphere by the year 2100 under one of the most severe IPCC forecasts (∼1000 µatm [21], Table 1). Hence, distinguishing anthropogenically driven changes in water column carbonate chemistry from natural variations could be far more challenging in reef environments than in the open ocean [22]. These observations, combined with more recent experimental work, have also led to the suggestion that biologically driven changes in water column carbonate chemistry could be providing a natural feedback on rates of calcification [23]. Because of the potential importance of this hypothesis, recent field studies have begun to more carefully examine the relationship between rates of net community calcification and in situ carbonate chemistry versus other important variables such as light, temperature, net production, and nutrients [23]–[26].

**Table 1:** Observed ranges in the *p*CO_2_ of reef waters worldwide (µatm).

Site	min	max	Ref.
Enewetak, Marshall Islands	150	320	[20]
Siraho Reef, Ishigaki, Ryukyus	160	520	[88]
Tiahura Reef, Moorea, Fr. Polynesia	240	420	[57]
Yonge Reef, north GBR	250	700	[57]
Bora Bay, Miyako, Ryukyus	200	550	[56]
Rukan-Sho, Okinawa, Ryukyus	40	900	[89]
Grand Bahama Bank, Bahamas	300	380	[28]
Hog Reef, Bermuda	320	560	[23]
Kaneohe Bay, Oahu, Hawaii	250	680	[25]
Lady Elliot Island, south GBR	100	1300	[30]
Sandy Bay, Ningaloo, W. Australia	220	440	[24]

Although there has been a recent renewed interest in how benthic reef metabolism alters the carbonate chemistry of coral reef waters [27]–[30], comparatively little attention has been given to the role that geomorphology and hydrodynamics play in modulating biologically driven changes in water chemistry. This is surprising since it is well-known that changes in water chemistry are as dependent on the residence time or ‘age’ of water masses as they are on the average rates of inorganic and biological reactions [31]. The residence time of reef waters change with location and time depending on the overall morphology of the reef system as well as on the hydrodynamic forcing mechanisms driving circulation [32], [33]. Circulation in most shallow reef systems is predominantly wave-driven (depending upon exposure), but it is also influenced by the tides and in some cases wind [33]–[35]. While some assessment of local hydrodynamics has always been critical for estimating in situ rates of benthic community metabolism, in many instances precise measurements of water chemistry are combined with only very rough estimates of flow speed, direction, and/or water residence time. This makes it difficult to properly assess the true uncertainty in derived rates of benthic metabolism (e.g., net production and net calcification).

Field studies of reef water chemistry and derived rates of benthic community metabolism are thus complicated by ever-changing circulation patterns and water residence times. The application of numerical hydrodynamic models within coral reef systems has provided us with one means to overcome this limitation [34]–[36]. These models essentially divide an entire reef system into many grid cells (∼10^5^ to 10^6^) that act as individual ‘control volumes’ through which net inputs and outputs of mass and momentum are budgeted. More recently, the inclusion of benthic metabolism within such models have provided the additional means for modeling spatial and temporal changes in nutrient concentrations and carbonate chemistry within coral reef systems [33], [37]. The application of coupled hydrodynamic-biogeochemical models has thus provided us with a powerful new tool for studying oceanic and atmospheric forcing of reef biogeochemical cycles.

While prior efforts have used numerical models for the study of specific reef systems, the range of biogeochemical changes that an individual reef system can exhibit is still constrained by its particular geomorphology, benthic composition, and by the specific oceanic and atmospheric conditions driving internal circulation and exchange with offshore waters. This constrained range of observable behavior is often further limited by the finite duration of many field studies (generally weeks or less). Long-term monitoring efforts can overcome some of these time-dependent limitations, but such programs generally come at great cost and are therefore limited in number. To overcome the limitation of examining a single reef system under pre-defined conditions, we developed a flexible model reef system with simple but representative geomorphologies and rates of benthic metabolism that can be subject to a wide range of hydrodynamic forcing. We show that this approach offers the same level of focus and control in system-scale studies that experimental mesocosms accomplish in organism-scale studies. Both approaches complement more realistic but less-controlled field studies by exploring the effects of specific forcing variables on the response of a system (or organism) over a broader range of controlled conditions. If designed properly, the hydrodynamics and biogeochemistry of these experimental idealized reefs can be remarkably similar to real reef systems [37], [38].

The present study is aimed at exploring how hydrodynamic factors (wave forcing and sea level), benthic metabolism, and reef geomorphology act in combination to fundamentally influence water column carbonate chemistry across representative wave-driven reef systems under a realistic range of input variables. The wealth of data on benthic net production, benthic net calcification, wave transformations, circulation, and morphology already available in the literature provide us with ample means to constrain our model domain within realistic limits (Tables 2 and 3). In this paper we first develop a set of relatively simple analytical equations to describe how changes in water column carbonate chemistry functionally depend on rates of benthic metabolism, wave forcing, sea level, and geomorphology using a simplified one-dimensional framework. Second, we simulate changes in wave heights, currents, and carbonate chemistry across a fully three-dimensional wave-driven reef-lagoon system under a wide range of physical and biogeochemical conditions. Third, we test the ability of the simple analytical model to replicate the behavior of our fully three-dimensional numerical model as well as to predict the variations in water column *p*CO_2_ that have been observed in natural reef systems. Finally, we discuss the utility of our simplified model to simulate larger reef systems as well as its implications for future studies of ocean acidification in coral reef systems.

**Table 2:** Daily rates of reef carbon metabolism worldwide.

Site	*P*	*R*	*G*_net_	*P*∶*R*	*G*_net_∶*P*	Ref.
Rongelap Atoll, French Polynesia	330	290	–	1.14	–	[19]
Enewetak Atoll, Marshall Islands	830	830	110	1.00	0.13	[18], [20], [90]
Fringing reef, Kauai, Hawaii	660	630	–	1.05	–	[17]
Laccadives, Northern Indian Ocean	520	210	–	2.48	–	[91]
One Tree Island, Central GBR	610	590	130	1.03	0.21	[92]–[94]
Guam, Marianas Islands	600	550	–	1.09	–	[95]
Lizard Island, Northern GBR	620	610	110	1.02	0.18	[96], [97]
French Frigate Shoals, NWHI	540	330	180	1.64	0.33	[98]
Rib Reef, Central GBR	600	670	100	0.90	0.17	[99]
Tulear, Madagascar	1580	920	50	1.72	0.03	[100]
Tiahura Reef, Moorea, Fr. Polynesia	660	610	170	1.15	0.26	[101], [102]
Yonge Reef, Northern GBR	1280	1250	250	1.02	0.20	[101]
Soraho, Ishigaki Island, Ryukyus	150	100	20	1.50	0.13	[88]
Bora Bay, Miyako Island, Ryukyus	720	530	50	1.36	0.07	[56], [103]
St. Gilles Reef, La Reunion Island	640	660	160	0.97	0.25	[104]
St. Leu Reef, La Reunion Island	1540	1280	150	1.20	0.10	[104]
Nature Reserve Reef, Gulf of Aqaba	340	300	50	1.13	0.15	[26], [105]
Kaneohe Bay barrier reef, Hawaii	670	590	250	1.17	0.36	[25], [44], [45]
Sandy Bay, Ningaloo, W. Australia	1230	1190	200	1.03	0.16	[24]
Coral Bay, Ningaloo, W. Australia	–	–	120	–	–	[33]
**Mean**	**740**	**640**	**130**	**1.24**	**0.18**	
*Std. Dev.*	*390*	*340*	*70*	*0.38*	*0.09*	
*Std. Err.*	*90*	*80*	*20*	*0.09*	*0.02*	
**Median**	**640**	**610**	**130**	**1.09**	**0.17**	
**Model Central Case**	**660**	**610**	**130**	**1.08**	**0.20**	***This study***

Daily integrated rates of community production (*P*), respiration (*R*), and net calcification (*G*_net_) published for reef flat communities across the Indo-Pacific over the last 60+ years. Also shown are the ratios of production to respiration and production to net calcification. Data presented for systems from which there were multiple sources were averaged. ‘–’ means no data available. Data for Rongelap through Tulear were taken from Kinsey 1985 [39].

**Table 3:** Offshore wave heights, transport, and geomorphology of reefs worldwide.

Site	*H*_o_	*U*_r_	*q*_r_	*h*_r_	*L*_r_	*W*_r_	*h*_c_	*L*_lag_	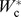	Refs
Kaneohe Bay, Oahu, Hawaii	2.0	0.13	0.26	2.0	1500	5500	8	1000	0.15	[106] [40]
Sandy Bay, Ningaloo Reef	1.5	0.10	0.13	1.3	450	3000	4	500	0.18	[37] [35]
Coral Bay, Ningaloo Reef	2.0	0.20	0.20	1.0	500	1100	5	700	0.15	[33]
Moorea, French Polynesia	1.5	0.18	0.31	1.7	450	3750	10^a^	500	0.05	[107] [43]
Bora Bay, Miyako, Ryukyus	1.0	0.25	0.25	1.0	250	600	2	500	0.15	[51]
Heron Island, South GBR	3.5	–	–	2.0	700	8000	3	1000	0.20	[41]
John Brewer Reef, Central GBR	0.3	0.08	0.08	1.0	400	5000	8	2000	0.10	[108]
Rib Reef, Central GBR	–	0.15	0.15	1.0	400	500	10	500	0.30	[109] [99]
Yonge Reef, North GBR	2.0	–	–	0.8	500	5800	35^c^	–	0.25	[110]
Abore Reef, New Caledonia	1.0	0.20	0.16	0.8	150	24000^b^	18^b^	20000^b^	0.10	[111]
**Mean**	**1.6**	**0.16**	**0.19**	**1.3**	**530**	**3700**	**6.3**	**840**	**0.16**	
*Std. Dev.*	*0.9*	*0.06*	*0.08*	*0.5*	*380*	*2600*	*9.8*	*500*	*0.07*	
*Std. Err.*	*0.3*	*0.02*	*0.03*	*0.1*	*120*	*900*	*3.5*	*200*	*0.02*	
**Median**	**1.5**	**0.17**	**0.18**	**1.0**	**450**	**3800**	**6.5**	**600**	**0.15**	
**Model Central Case**	**1.5**	**0.18**	**0.18**	**1.0**	**500**	**4000**	**6.0**	**700**	**0.15**	***This study***

**Figure 1:**
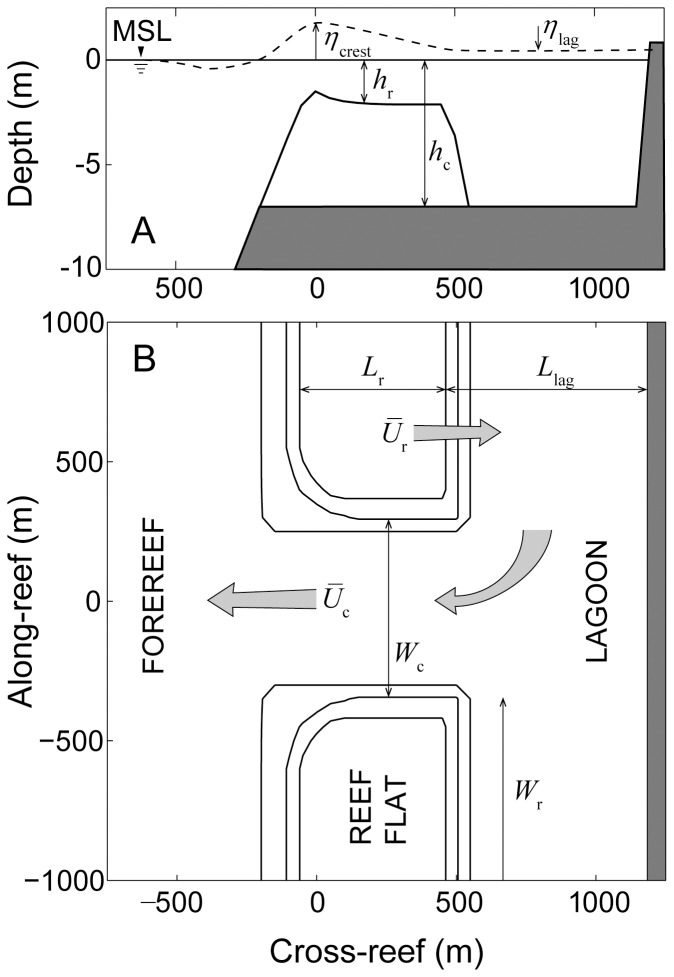
Schematics of the coastal reef-lagoon system. (A) side-view depth profile of the reef flat and (B) top-view of part of the domain containing sections the forereef, reef flat, lagoon and channel. The ratio of vertical to horizontal scale in (A) is 75∶1. The main reef structures in (B) are outlined by the 1.5-m, 3-m, and 6-m isobaths. Dark grey regions represent land or solid reef basement. Light grey arrows represent the general direction of wave-driven circulation. The reference origin set at the intersection of the reef crest line and mid-channel is provided only to illustrate the scale of the reef features. All morphological dimensions shown reflect those of the central model case; however, an extra +1 m of sea level has been added to (A) to better illustrate profiles of reef flat depth and cross-reef setup. See Background and Table 4 for additional description of all variables shown.

Summary of offshore wave heights, flow, and reef morphology parameters reported from hydrodynamic studies of reefs across the Indo-Pacific and augmented by the additional analysis of satellite imagery provided by Google Earth. Notes: (a) the effective channel depth for Moorea was set at 10 m to reflect that setup in the channel was roughly one-half of that at the reef crest even though maximum channel depths can exceed 30 m [43], (b) the reef flat width, depth of channel, and lagoon width for Abore Reef were considered extreme and excluded from the calculation of the group statistics, (c) the extreme depth of the channel adjacent to Yonge Reef is relevant to exchange for an entire section of the north GBR lagoon, not just local flow across Yonge Reef and therefore excluded from the calculation of the group statistics (see Discussion). See Background, Fig. 1, or Table 4 for additional description of variables shown.

### Background

#### Prior studies of reef metabolism and wave driven circulation

In a seminal review of reef community carbon metabolism, Kinsey 1985 [39] found that rates of daily benthic community gross production (*P*), respiration (*R*), and net calcification (*G*_net_) for entire reef flats tended to cluster around typical or ‘standard’ values of 580, 580, and 110 mmol C m^−2^ d^−1^, respectively. These rates were found to be largely independent of latitude and longitude (at least across the Indo-Pacific) despite seasonal and spatial variations in light, ocean sea surface temperature, and carbonate mineral saturation state; although seasonality in *G*_net_ appeared to be stronger in higher-latitude reefs [39]. More recent data collected over the 30 years following the Kinsey 1985 review has not fundamentally changed this general assessment of reef flat metabolism (Table 2). However, a review of the larger cumulative data set indicates that 1) rates of benthic reef carbon metabolism fall within a range that is a factor of two lower or higher than the ‘standard’ or median rates (*P*≈300 to 1200 vs. 640 mmol C m^−2^ d^−1^ and *G*_net_≈50 to 250 vs. 130 mmol C m^−2^ d^−1^, Table 2), and 2) shallow reef communities are modestly net autotrophic on average (i.e., *P*∶*R* ∼1.1, Table 2).

Although there have been fewer studies of wave transformations and/or wave-driven circulation across shallow reef systems, it is nonetheless clear that the geomorphology and hydrodynamic forcing of many reef systems also fall within well-defined ranges (Table 3). The archetypal or ‘standard’ reef (Fig. 1) has a reef flat that is ∼500 m long 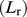 and ∼1 m deep at mean sea level 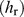, is backed by a lagoon that is anywhere from 500 m to ∼20 km long 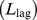, has channels that are 10% to 20% the width of the reef flat 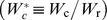 and 2 to ∼20 m deep 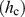, and is exposed to offshore significant wave heights of between 1 and 2 m (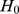). As a result, cross-reef flow speeds 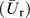 are generally between 0.1 to 0.2 m s^−1^ resulting in cross-reef transports 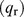 that are between 0.1 and 0.2 m^2^ s^−1^ 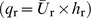.

#### Theory

Lowe et al. 2009 [40] showed that a simple one-dimensional (cross-reef) model captures the essential dynamics of wave-driven circulation in shallow coastal reef systems (i.e., forcing and response) despite the more complex, two-dimensional (horizontal) structure in the circulation exhibited by real reef systems [34]. Circulation in wave-exposed reef systems is ultimately controlled by wave breaking in the shallow surf zone which, in turn, causes the mean sea level at the reef crest to increase relative to offshore. This wave setup height 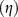 in turn drives the flow of water across the shallow reef flat, through the lagoon, and out the nearest channel (Fig. 1). Gourlay and Colleter 2005 [41] developed a semi-empirical formulation to describe the maximum wave setup at the reef crest 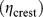 as a function of incident wave power which can be approximated by the following relationship:
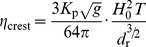
(1)where 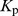 is the empirical reef profile factor that depends on the reef slope (∼0.8 for a near-vertical reef face, 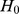 is the offshore wave height, *T* is the wave period, 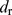 is the total depth of the reef flat equal to the sum of the reef flat depth at mean sea level and the reef flat setup 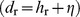, and *g* is the gravitational acceleration constant (see Appendix S1). The 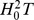 term in the second numerator is proportional to the shoreward flux of surface wave energy [42].

Bottom friction causes setup to decrease across the reef flat and channel such that
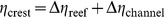
(2)assuming offshore mean sea level is zero 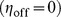. Because flow speeds in the channel are generally higher than on the reef flat, reef channels can potentially provide enough frictional resistance to the wave-driven flow such that setup in the lagoon becomes a significant fraction of the setup at the reef crest [40], [43]. Assuming a balance of both mass and momentum across the reef and channel, we can derive an expression for the cross-reef transport as a function of the offshore wave conditions and the combined morphology of the reef flat and channels:
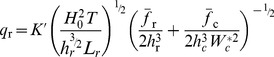
(3)where 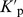 is equal to 
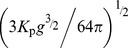
, and 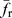 and 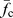 are the bottom friction coefficients corresponding to the depth-averaged flow speed over the reef flat and in the channel, respectively (see Appendix S2). 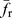
and 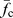 are primarily a function of physical bottom roughness expressed in terms of a hydraulic roughness length scale 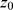 and the local water depth, but are also subject to wave-current interactions; especially within the wave-exposed channel (see Appendix S3).

Assuming a vertically well-mixed water column, the change in concentration of a reactive species with space 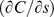 and time 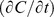 along a flow streamline 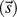 is primarily a function of the benthic flux (*J*), the water depth, and the horizontal transport
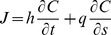
(4)Prior studies have shown that the advective term (second term, right-hand side) is generally much greater than the time-dependent term (first term, right-hand side) for water crossing the reef flat [24], [44], [45].
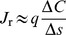
(5)Thus, we can re-formulate the change in reactive species concentration across the reef flat 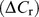 as a function of the reef flat length 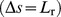 and the cross-reef transport, i.e.
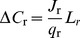
(6)Combining Eq. 3 and Eq. 6 we get the following relationship describing the change in reactive species concentration across the reef flat as a function of the benthic flux, incident wave forcing, reef morphology, and frictional roughness:
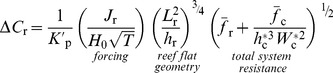
(7)where 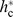 is the ratio of the channel depth to the reef flat depth 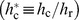
and all other variables are defined as before. Eq. 7 can be expressed more simply as:
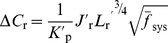
(8)where
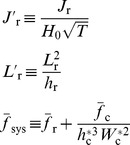
(9)
Eq. 8 indicates that changes in the concentration of a reactive species depend on three fundamental properties: 1) a forcing factor determined by the ratio of benthic metabolism or bottom flux to the square root of the onshore wave energy flux 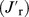, 2) a reef flat geometry factor determined by the length and depth of the reef flat 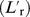, and 3) the total resistance of the reef system to wave-driven flow 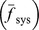. The role of each of these factors can be explained as follows. Greater bottom fluxes will drive greater changes in water chemistry while stronger wave forcing will increase transport and decrease the contact time of water with the reactive benthos thus reducing changes in concentration. The length and depth of the reef flat are important for several reasons. Firstly, changes in water chemistry from variable bottom fluxes become increasingly diluted as the depth of the water column increases. Secondly, the longer the reef flat, the greater the time for water to interact with the reef (Eq. 6). Thirdly, as the length of the reef flat increases, the cross-reef gradient in setup across the reef becomes weaker 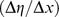, and cross-reef transport decreases (Eq. 3); thus, increasing the change in concentration per unit length of reef flat (Eq. 6). Fourthly, the greater the reef flat depth, the lower the maximum setup at the reef crest (Eq. 1), and the weaker the cross-reef transport; a result which also increases the change in concentration per unit length of reef flat (Eq. 6). The total frictional resistance of the reef system controls the rate of volume transport through the reef system under a given set of wave conditions. It is not only a function of the frictional roughness of the reef flat and channels, but also a function of the ratios of the channel depth to reef depth and the channel width to reef width as well. As the relative depth and/or width of the channel decrease (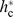 or 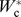), the frictional resistance generated in the channels can become a significant impediment to overall wave-driven circulation. This results in longer water residence times and consequently larger changes in water chemistry (Eq. 7).

## Methods

We define a central model case based on median and average rates of benthic metabolism, morphological dimensions, and wave forcing reported in the literature (see Tables 2 and 3). We assume that hourly rates of gross primary production, respiration, and net calcification are constant to some finite depth, and then decrease exponentially with depth proportional to the attenuation of light [33], [46]. Next, we explore the sensitivity of the idealized reef hydrodynamic-biogeochemical model to changes in 1) the ratio of benthic metabolism to wave forcing, 2) the geometry of the reef flat, 3) the depth and width of the channels connecting the lagoon and ocean via their impact on total system frictional resistance, and 4) sea level. Exploring the influence of each of these factors often involves more than one variable (Table 4). In all model simulations (except two) we varied only one of the ten possible input variables choosing a perturbation-response approach to evaluating model sensitivity in order to keep the number of simulations tractable and to simplify the analysis of our results. These individual variables included *H*_0_, *P*, *G*_net_, *P*_lag_, *h*_r_, *L*_r_, *L*_lag_, *h*_c_, *W*_c_, and the offshore sea level 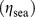.

**Table 4:** Variables and constants used in this paper.

Variable	Units	Description
*P*	mmol C m^−2^ d^−1^	Daily reef gross primary production
*P*_lag_	mmol C m^−2^ d^−1^	Daily lagoon gross primary production
*R*	mmol C m^−2^ d^−1^	Daily reef respiration
*G*_net_	mmol C m^−2^ d^−1^	Daily reef net calcification
*p*	mmol C m^−2^ hr^−1^	Hourly gross primary production
*p*_max_	mmol C m^−2^ hr^−1^	Maximum hourly gross primary production
*r*	mmol C m^−2^ hr^−1^	Hourly respiration
*np*	mmol C m^−2^ hr^−1^	Hourly net production
*g*_net_	mmol C m^−2^ hr^−1^	Hourly net calcification
*d*_sed_	mmol C m^−2^ hr^−1^	Sediment dissolution rate
TA	µeq kg^−1^	Total Alkalinity
DIC	µmol kg^−1^	Dissolved Inorganic Carbon
*p*CO_2_	µatm	Partial pressure of dissolved carbon dioxide
Ω	–	Carbonate mineral saturation state (calcite or aragonite)
Ψ	–	Molar ratio of CO_2_ released from calcification
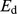	µmol m^−2^ s^−1^	Instantaneous downwelling planar PAR irradiance
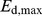	µmol m^−2^ s^−1^	Maximum downwelling planar PAR irradiance
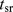	hr	Time of sunrise
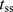	hr	Time of sunset
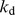	m^−1^	Light attenuation coefficient
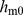	m	Depth of constant metabolism
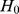	m	Offshore significant wave height
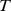	s	Offshore peak wave period
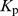	–	Wave power transmission parameter
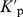	–	Modified wave power transmission parameter
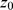	m	Roughness length scale
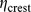	cm	Wave setup at the reef crest
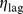	cm	Wave setup in the lagoon
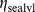	cm	Added sea level above mean sea level
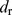	m	Reef flat depth (including waves and tides)
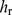	m	Reef flat depth at mean sea level
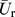	m s^−1^	Depth-averaged current
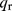	m^2^ s^−1^	Depth integrated cross-reef transport
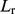	m	Reef flat length
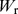	m	Reef flat width
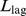	m	Lagoon length
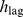	m	Lagoon depth
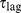	hr, d	Lagoon residence time
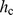	m	Channel depth
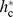	–	Relative channel depth
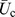	m s^−1^	Depth-averaged channel current
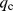	m^2^ s^−1^	Depth-integrated channel transport
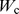	m	Channel width
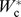	–	Relative channel width
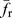	–	Depth-averaged reef flat current drag coefficient
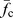	–	Depth-averaged channel current drag coefficient
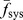	–	Total reef system frictional resistance
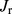	mmol m^−2^ hr^−1^	Rate of benthic reef flat metabolism
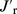	mmol m^−3^ hr^−1.5^	Ratio of reef flat metabolic forcing to wave forcing
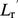	m	Reef flat geometry factor

‘–’ represent dimensionless variables.

### Numerical Model

The hydrodynamic and biogeochemical processes were simulated using the three-dimensional ocean circulation model ROMS (Regional Ocean Modeling System, version 3.3) [47] two-way coupled to a spectral wave model SWAN (Simulating WAves Nearshore, version 40.51) [48], based on recent nearshore routines implemented in ROMS [49]. The water column is divided into 4 vertical layers. A higher vertical resolution is not needed as the water column in the shallow reef area is assumed to be well-mixed [37]. A southward offshore boundary current of 0.25 m s^−1^ is chosen to simulate offshore momentum boundary conditions similar to those both observed and used in prior modeling studies [32], [36]; however, subsequent simulations indicated that model results were not sensitive to the offshore current speed (see Tables S1 through S5). The less important processes of local wind wave generation, nonlinear wave-wave interaction, wave-current refraction, and dissipation due to white capping in SWAN were turned off [34]. Directional wave spectra were prescribed at the western (offshore) boundary as well as the offshore section of the northern and southern boundaries using time-varying significant wave heights, peak wave periods and peak wave directions, by assuming a standard JONSWAP (Joint North Sea Wave Project) frequency distribution, 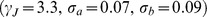 and a cosine to the power 5 directional distribution. The wave direction is from the west (270°) in all simulations. Depth-limited wave breaking was modeled with a breaking coefficient of 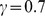, a value typically used in reef systems [34], [50], [51]. While there are three commonly used bottom boundary models to calculate wave-current interaction within ROMS, the Soulsby 1995 [52] model was chosen as it is computationally more efficient under a wide range of wave and current flow combinations and yet no less accurate than other models [37]. All model simulations were conducted in parallel using Message Passing Interface (MPI) on a supercomputer (iVEC; http://ivec.org.au/) using 132 processors.

**Figure 2:**
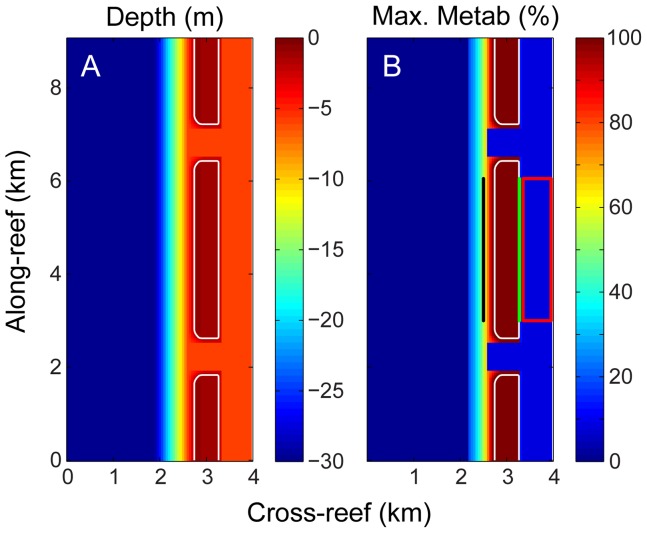
Bathymetry and benthic metabolism for the central model case. (A) Bathymetry and (B) rate of hourly metabolism (*p*, *r*, *g*_net_) as a percentage of the maximum rate for the entire reef system. In panel B the forereef and backreef transects defined in later analyses are indicated by the heavy black and green lines, respectively; while the lagoon zone defined in later analyses is indicated by the red box. The values shown along the *x* and *y* axes are only to illustrate the scale of the model domain.

The model domain consisted of one complete shallow reef flat bounded by a channel and one-half a reef flat to the north and to the south with a lagoon to the east (Fig. 2a) based on the design of Lowe et al. 2010 [38]. All model reefs reached a minimum depth of 0.5 m at the reef crest and then declined exponentially toward the back such that the average depth across the reef flat was equal to the target reef flat depth of 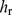 and the depth of the lagoon was always set equal to the depth of the channel 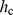 (Table 4, Fig. 1a). The combined widths of the reef flat and channels were kept constant and equal to 4600 m for all simulations. This means that as the channel width 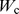 increased, the reef width 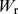 decreased such that 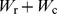 was equal to 4600 m in all simulations (Fig. 1b). This resulted in a model domain which was ∼9 km long in the along-reef (north-south) direction and ∼4 km in the cross-reef (east-west) direction (Fig. 2a). We needed a relatively small spatial resolution (50 m) to properly resolve changes in wave height, setup, and currents across the shallow forereef and reef flat [33], [35], [40]. Thus, to keep the model simple and tractable, we limited our simulations to lagoons that were 1500 m long 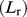 or less. The forereef slope was kept constant and equal to 1∶30 for all simulations; a value we chose to be intermediate between steep-faced reefs (1∶20 [41]) and more gently sloped reef fronts (1∶40 [40]). The maximum depth of the domain was limited to 30 m, since prior work has shown that chemical reactions at these depths in the presence of an alongshore current have a negligible influence on changes on water column chemistry across coastal reef-lagoon systems [33]. Finally, a roughness length scale 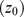 of 0.03 m was chosen to represent the reef flat and forereef and 0.015 m for the lagoon to reproduce quadratic bottom drag coefficients similar to those observed in prior studies (0.01 to 0.03 [33], [35], [40]).

### Biogeochemical Forcing

Hourly rates of gross production (*p*) and net calcification (*g*_net_) were constrained to equal daily integrated rates over a 24-hour period:
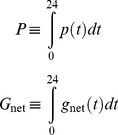
(10)(see Appendix S4). Hourly rates of respiration were assumed to be constant throughout the day
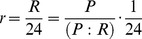
(11)Finally, hourly rates of net community production (*np*) were calculated as the difference between 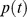
and *r*

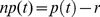
(12)For the central model case we further assumed that the bottom of the lagoon and channels were comprised of sand communities whose daily gross production, respiration, and net calcification were 80, 70, and 20 mmol C m^−2^ d^−1^, respectively [39], [53]–[55]; however, we ran additional simulations to examine the effect of higher rates of lagoon metabolism on changes in carbonate chemistry (*P*_lag_ = 330 mmol C m^−2^ d^−1^ and 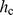 = 3 and 6 m, [39], [56]).

Local benthic fluxes of Total Alkalinity (TA) and Dissolved Inorganic Carbon (DIC) were then calculated from hourly rates of *g*_net_ and *np* as
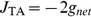
(13)

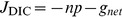
(14)where negative fluxes represent net removal of TA or DIC from the water column [20]. Given that the residence times of waters within the reef flat, lagoon, and channel were always less than 24 hours in all simulations, that water column *p*CO_2_ varied between undersaturated and oversaturated conditions with respect to the atmosphere over a diurnal cycle, and that air-sea CO_2_ fluxes are generally very small relative to the combined influence of benthic net production and calcification (<1% [20], [57]); we ignored the air-sea exchange of CO_2_ in the present model as it would have a negligible effect on simulated changes in carbonate chemistry on these time scales (∼1 day or less).

Offshore waters were assumed to be at a uniform temperature of 25°C and salinity of 35, in equilibrium with the atmosphere (*p*CO_2_ = 390 µatm), and having a sea surface TA typical of tropical surface waters worldwide (2300 µeq kg^−1^ [58]). Offshore DIC and all other carbonate chemistry parameters were calculated from these defined input conditions using the CO2SYS program [59] based on pre-defined dissociation and solubility constants [60]–[63]. Preliminary simulations indicated that differences in the model predicted transport and carbonate chemistry parameters simulated on a 50-m resolution grid and those simulated on a 25-m resolution grid differed by less than a few percent; therefore, all subsequent simulations were run on a 50-m grid. Preliminary simulations also indicated that changes in carbonate chemistry across the domain reached equilibrium to within less than ∼2 µeq kg^−1^ d^−1^ of TA, ∼1 µmol kg^−1^ d^−1^ of DIC, and less than ∼3 µatm d^−1^ of *p*CO_2_ after the first 24 hours of simulation time. Therefore, all simulations were run for 2 days (simulation time) in order for diurnal changes in water column carbonate chemistry to reach an approximate steady state (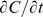
<1% d^−1^) under the imposed constant hydrodynamic and forcing and diurnal variations in benthic metabolism.

## Results

### Three-dimensional simulations

**Figure 3:**
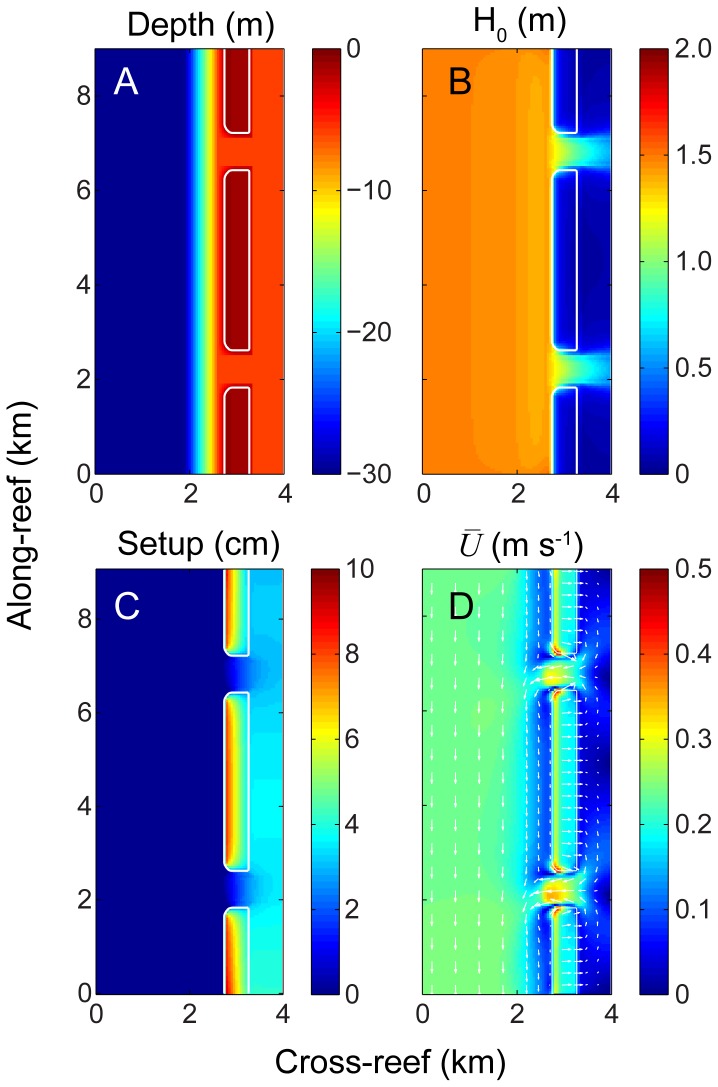
Hydrodynamic simulation results. Variations in A) bathymetry, B) significant wave height, C) setup, and D) depth-averaged flow speed and direction for the central case.

Wave heights decreased rapidly near the reef crest due to depth-limited breaking with some wave energy penetrating into the deeper channels (Fig. 3b). This pattern of wave dissipation led to maximum wave-induced setup near the shallow reef crest. Wave setup then declined sharply across the reef flat before reaching levels within the lagoon that were less than on the reef flat, but still higher than offshore (Fig. 3c). The resulting spatial distribution of the wave setup drove a general pattern of circulation whereby waters moved lagoonward across the reef flat and seaward out the channels; a pattern consistent with numerous prior observations of wave-driven circulation in coral reefs [33]–[35], [40], [43], [64]. Water flowing out of the main channels was quickly entrained by the southward alongshore current within ∼1 km offshore of the channel entrance (Fig. 3d). For reference, the exact output of key hydrodynamic variables (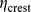,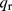,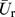,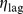,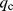,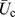, and average significant wave heights in the channel 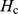) for each of the 33 simulations are provided in Table 5.

**Table 5:** Hydrodynamic data from all simulations including setup at the reef crest, cross-reef transport and currents observed at the back of the reef flat, and setup in the lagoon, as well as average transport, currents, and average significant wave heights within the channel.

	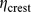	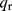	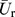	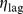	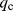	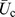	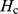
Simulation	cm	m^2^ s^−1^	m s^−1^	cm	m^2^ s^−1^	m s^−1^	m
Central Case	8.0	0.21	0.18	3.6	−1.8	−0.30	1.09
*H*_0_ = 0.5 m	1.2	0.09	0.08	0.6	−0.8	−0.13	0.42
*H*_0_ = 1 m	4.2	0.15	0.14	1.7	−1.3	−0.21	0.77
*H*_0_ = 2 m	11.9	0.24	0.22	6.1	−2.2	−0.37	1.38
*H*_0_ = 3 m	19.5	0.29	0.26	12.8	−2.9	−0.49	1.85
*h*_r_ = 0.5 m	10.3	0.08	0.17	2.5	−1.0	−0.17	1.08
*h*_r_ = 0.7 m	9.2	0.13	0.18	3.1	−1.3	−0.22	1.08
*h*_r_ = 1.5	6.5	0.27	0.15	4.4	−2.1	−0.36	1.09
*h*_r_ = 2.0 m	5.8	0.28	0.12	4.5	−2.3	−0.38	1.09
*L*_r_ = 250 m	6.7	0.26	0.22	3.9	−2.1	−0.34	1.17
*L*_r_ = 500 m	8.6	0.17	0.15	3.9	−1.6	−0.27	1.00
*L*_r_ = 1000 m	9.1	0.14	0.13	3.8	−1.5	−0.26	0.92
*h*_c_ = 3 m	9.7	0.12	0.11	8.6	−1.1	−0.35	0.85
*h*_c_ = 4.5 m	8.4	0.18	0.16	5.6	−1.5	−0.33	1.01
*h*_c_ = 10 m	7.5	0.24	0.21	1.6	−2.2	−0.22	1.16
*W*_c_ = 200 m	9.2	0.14	0.13	7.6	−3.6	−0.61	0.90
*W*_c_ = 300 m	8.5	0.17	0.15	6.0	−2.9	−0.49	1.00
*W*_c_ = 450 m	8.2	0.19	0.17	4.5	−2.2	−0.37	1.06
*W*_c_ = 1200 m	7.7	0.22	0.20	2.4	−0.9	−0.15	1.11
*ç*_sea_ = +0.5 m	5.7	0.30	0.19	4.3	−2.4	−0.37	1.12
*ç*_sea_ = +1 m	4.5	0.36	0.17	4.3	−2.8	−0.40	1.15
*ç*_sea_ = +2 m	3.4	0.37	0.12	3.4	−3.0	−0.37	1.19
*ç*_sea_ = +4 m	1.5	0.23	0.04	1.4	−2.0	−0.20	1.25
*P* = 150	8.0	0.21	0.18	3.6	−1.8	−0.30	1.09
*P* = 330	8.0	0.21	0.18	3.6	−1.8	−0.30	1.09
*P* = 1000	8.0	0.21	0.18	3.6	−1.8	−0.30	1.09
*P* = 1500	8.0	0.21	0.18	3.6	−1.8	−0.30	1.09
*G*_net_ *∶P = 0%*	8.0	0.21	0.18	3.6	−1.8	−0.30	1.09
*G*_net_ *∶P = 40%*	8.0	0.21	0.18	3.6	−1.8	−0.30	1.09
*P*_lag_ = 330	8.0	0.21	0.18	3.6	−1.8	−0.30	1.09
*P*_lag_ = 330, *h*_c_ = 3 m	9.7	0.12	0.11	8.6	−1.1	−0.35	0.85
*U*_off_ = 0.125 m s^−1^	8.0	0.21	0.18	3.6	−1.7	−0.29	1.09
*L*_lag_ = 1500 m, *h*_c_ = 20 m	7.3	0.25	0.22	0.5	−3.5	−0.17	1.13

All data shown represent values averaged over line transects and zones identified in Fig. 2B.

**Figure 4:**
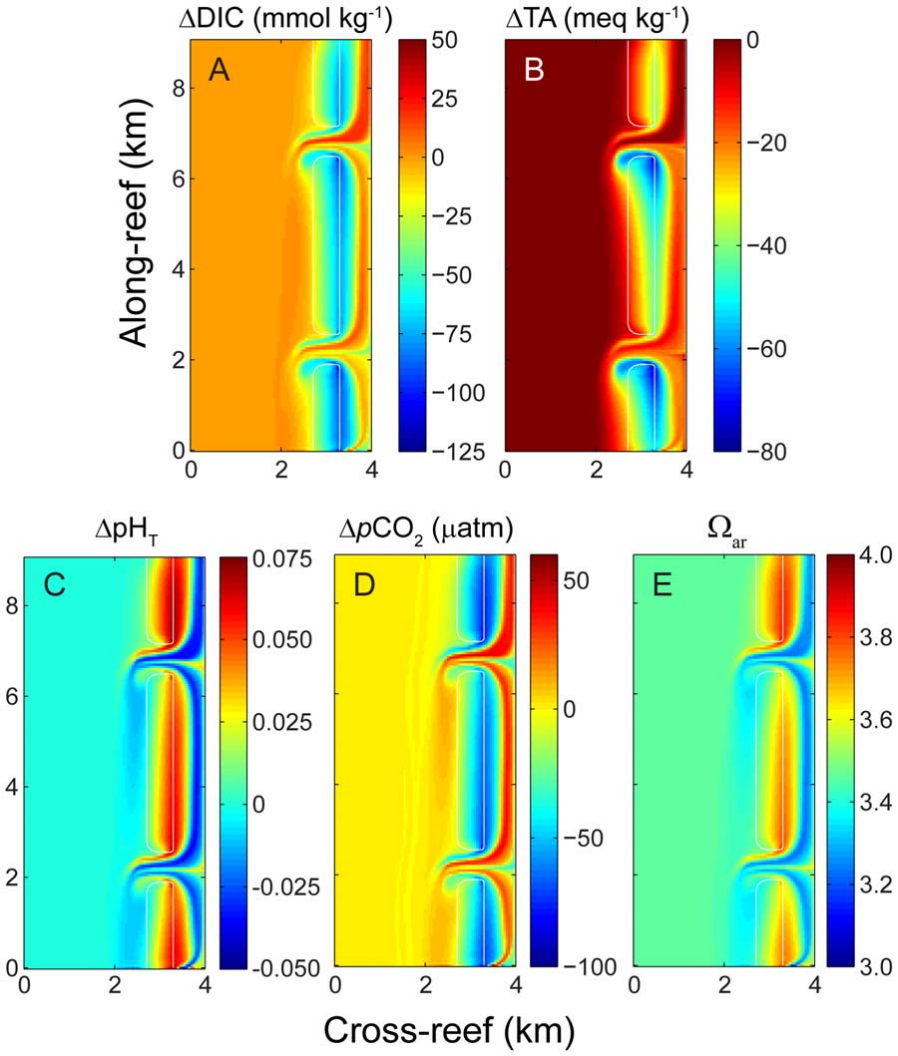
Spatial variation in carbonate chemistry at mid-day. Changes in A) dissolved inorganic carbon, B) total alkalinity, C) pH_T_, and D) water column *p*CO_2_ relative to offshore values as well as E) spatial variation in aragonite saturation state at mid-day for the central case.

Changes in TA, DIC, *p*CO_2_, and Ω_ar_ across the reef flat at mid-day (i.e., peak solar irradiance) are driven by their benthic uptake from net production and net calcification; behavior consistent with numerous observations reported in the literature (Fig. 4). Water in the lagoon and channels exhibited both high and low ΔTA, ΔDIC, Δ*p*CO_2_, and Ω_ar_ due to the confluence of water masses which had traversed the shallow reef flat at different phases of day and night. This general spatial pattern was similar for all carbonate chemistry parameters differing only in scale and sign (negative for ΔTA, ΔDIC, Δ*p*CO_2_, and positive for ΔpH and ΔΩ_ar_). Therefore, in the interest of economy, most of the results and discussion that follows will focus on variations in *p*CO_2_ since this is the primary variable of interest with regards to future changes in atmospheric composition. Nonetheless, time-elapsed movies demonstrating hourly changes in ΔTA, ΔDIC, Δ*p*CO_2_, ΔpH and ΔΩ across the entire reef domain over a 24-hr period are also provided (see Movies S1 through S5).

**Figure 5:**
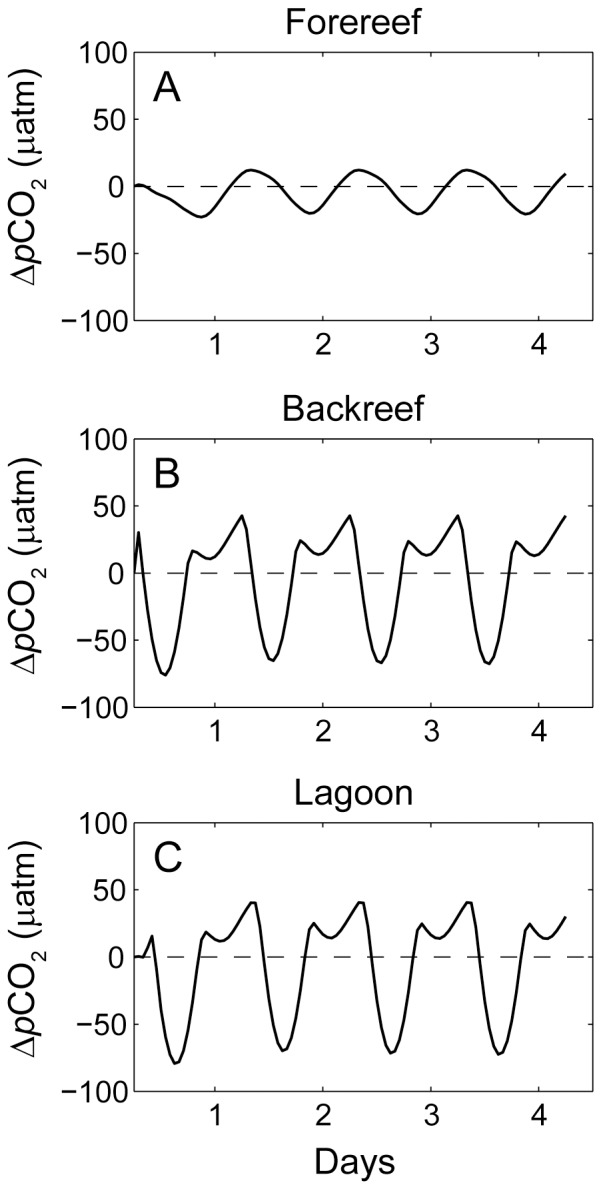
Diurnal variations in water column *p*CO_2_. Changes in dissolved *p*CO_2_ in waters on the A) forereef, B) backreef, and C) lagoon relative to offshore values for the central case. A four-day simulation was run for the central case and is shown here in its entirety solely to indicate the stability of the model simulation after just one day. For each location shown, the observations were recorded at the centermost point of the forereef and backreef transects as well as lagoon zone shown in Fig. 2B. The dashed line indicates no change in *p*CO_2_.

Diurnal changes in Δ*p*CO_2_ on the forereef appeared to follow quasi-sinusoidal variations (Fig. 5a), whereas diurnal variations in Δ*p*CO_2_ on the reef flat and in the lagoon exhibited more complex temporal behavior (Fig. 5b, 5c). This was largely due to the re-entrainment of channel outflow water into the cross-reef flow after exiting the channels and then moving southward across the forereef with the predominant alongshore current (Figs. 3d, 4, 5). Consequently, re-entrained low-Δ*p*CO_2_ water produced during the day damped maximum nighttime values resulting in truncated or ‘scalloped’ maxima. This same pattern was evident in the time series of ΔTA and ΔDIC (not shown). It is possible that this effect could be even more pronounced when alongshore currents, including the associated horizontal mixing, on the forereef are particularly weak. However, reducing the magnitude of the offshore current by one-half relative to the central case had only a modest effect on diurnal profiles of forereef water column carbonate chemistry. Furthermore, prior field observations (not shown) suggest that our reef-scale models if anything slightly overestimate the observed variability in the chemistry of forereef waters. This is likely the result of reef-scale models being unable to capture larger-scale hydrodynamic processes (e.g., those operating on the shelf) that may also contribute significantly to cross-shelf mixing and transport.

Since diurnal minima and maxima in carbonate chemistry parameters can be both complex and short-lived for many locations (on real reefs as well as on our model reef), we defined a statistically representative amplitude (*A*) of diurnal variation in a given carbonate chemistry parameter based on the temporal variance in that parameter value
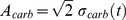
(15)where *carb* represents either TA, DIC, *p*CO_2_, pH, or Ω_ar_ and 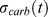 is estimated by the standard deviation of the depth-averaged parameter value at a given location from the mean over a 24-hour period. Thus, if the diurnal variation is quasi-sinusoidal, then the range between minima and maxima will be ∼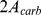, or roughly 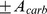 about the mean. We further defined 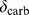 to represent the ‘net offset’ of TA, DIC, *p*CO_2_, pH, or Ω_ar_ in reef waters relative to offshore waters (Δ*carb*) averaged over a 24-hour period
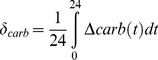
(16)Defining 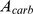 and 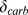 in Eqns. 15 and 16 allowed us to distil the four-dimensional output of our simulations (*x, y, z, t*) into two-dimensional variables that vary only with location (*x, y*) through depth- and time-averaging.

**Figure 6:**
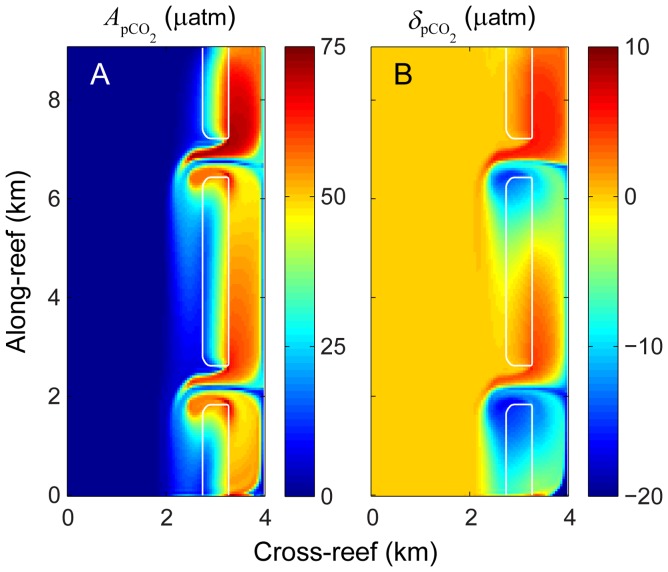
Diurnal and net changes in water column *p*CO_2_. A) Amplitude of diurnal *p*CO_2_ variation 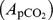
and B) net offset of *p*CO_2_ relative to offshore waters over a 24-hour period 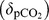 for the central model case.

Both 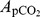 and 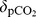 varied across the reef domain, increasing from the shallow forereef to the backreef and lagoon, and reaching maximum levels inside the channels (Fig. 6). Here we define the ‘forereef’ as the reef area seaward of the reef crest but excluding the outer channel, and ‘backreef’ as the point furthest lagoonward but still on the reef flat (see Fig. 2). The full output of 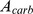 and 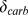 for TA, DIC, *p*CO_2_, pH, or Ω_ar_ in each of the three zones and for all 33 simulations are provided in Tables S1 through S5. As waters with altered chemistry exited the channel they became both entrained and diluted by the predominant current as it moved southward. This resulted in noticeable along-reef variations in water chemistry across the reef flat and lagoon. This entrainment effect was particularly pronounced at the boundaries between the reef flats and channels where lagoonward flow across the reef flat and seaward flow out the channels generated notable velocity shear. The re-circulation of waters around eddies generated by these flow features near the channel mouth caused local alterations in water chemistry that were higher than the surrounding reef flat (Fig. 6). Prior numerical studies have shown that these features are less pronounced under more realistically variable hydrodynamic forcing, i.e. as opposed to the stationary offshore wave forcing assumed in the present study. Therefore, for real reefs we expect these eddies to only influence variations in water chemistry at a local scale (100 s of m) near the edges of channel mouths. Regardless, we would still caution against deriving system-scale budgets from chemical measurements made near these hydrodynamically complex transitions.

**Figure 7:**
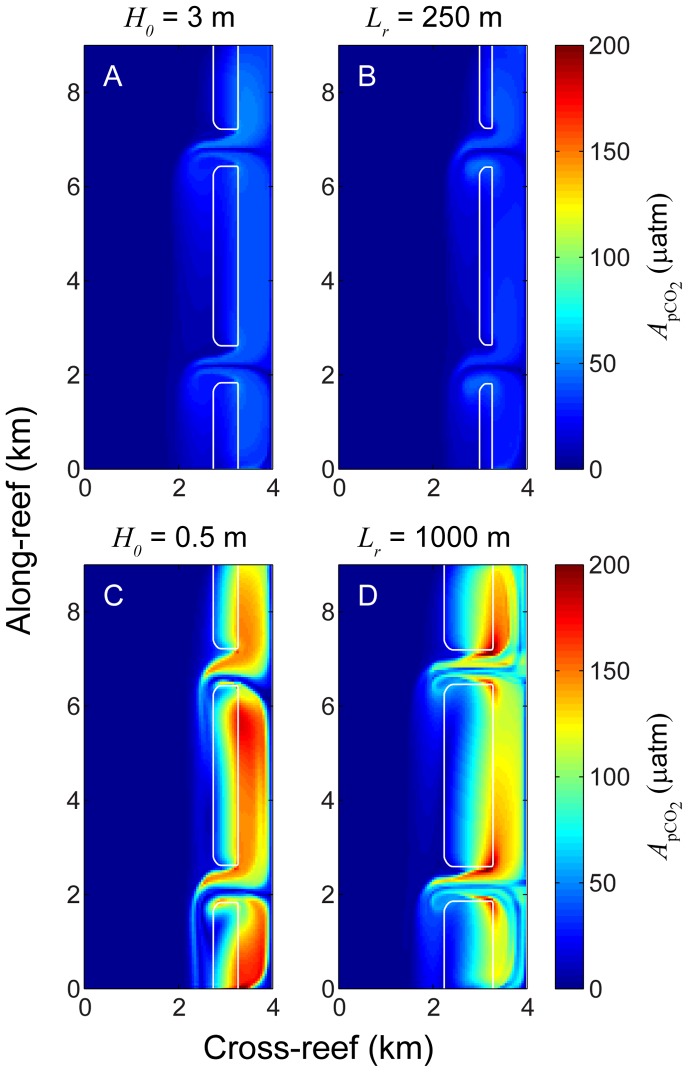
Weak and strong variation in *p*CO_2_. Amplitude of diurnal *p*CO_2_ variation 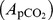
for simulations based on the following variations in offshore wave height and reef flat length: A) *H*_0_ = 3 m, B) *L*_r_ = 250 m, C) *H*_0_ = 0.5 m, and D) *L*_r_ = 1000 m. Simulations shown in A and B represent conditions resulting in relatively weak variations in carbonate chemistry while simulations C and D represent conditions resulting in relatively strong variations in carbonate chemistry.

Changes in water column *p*CO_2_ varied not only by location, but with the rate of metabolism, degree of wave forcing, and reef geomorphology as well. Sample plots of 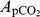 for select simulations resulting in both weak and strong variation in water column carbonate chemistry are provided as examples in Fig. 7. Higher offshore waves and/or lower rates of benthic metabolism resulted in weaker variations in carbonate chemistry, whereas lower offshore waves and/or higher rates of benthic metabolism resulted in stronger variations in carbonate chemistry. In the interest of distilling this two-dimensional data even further, we next calculated representative 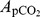 and 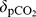 for key sites within the reef domain by averaging 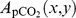 and 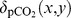 across forereef and backreef transects as well as across most of the lagoon (Fig. 2b). This is because the general pattern of spatial variations in 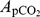 and 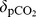 across all other simulations were similar to that exhibited by the central model case (Figs. 5, 6); i.e., the spatial variation in 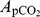 and 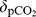 differed mainly in magnitude rather than in structure.

**Figure 8:**
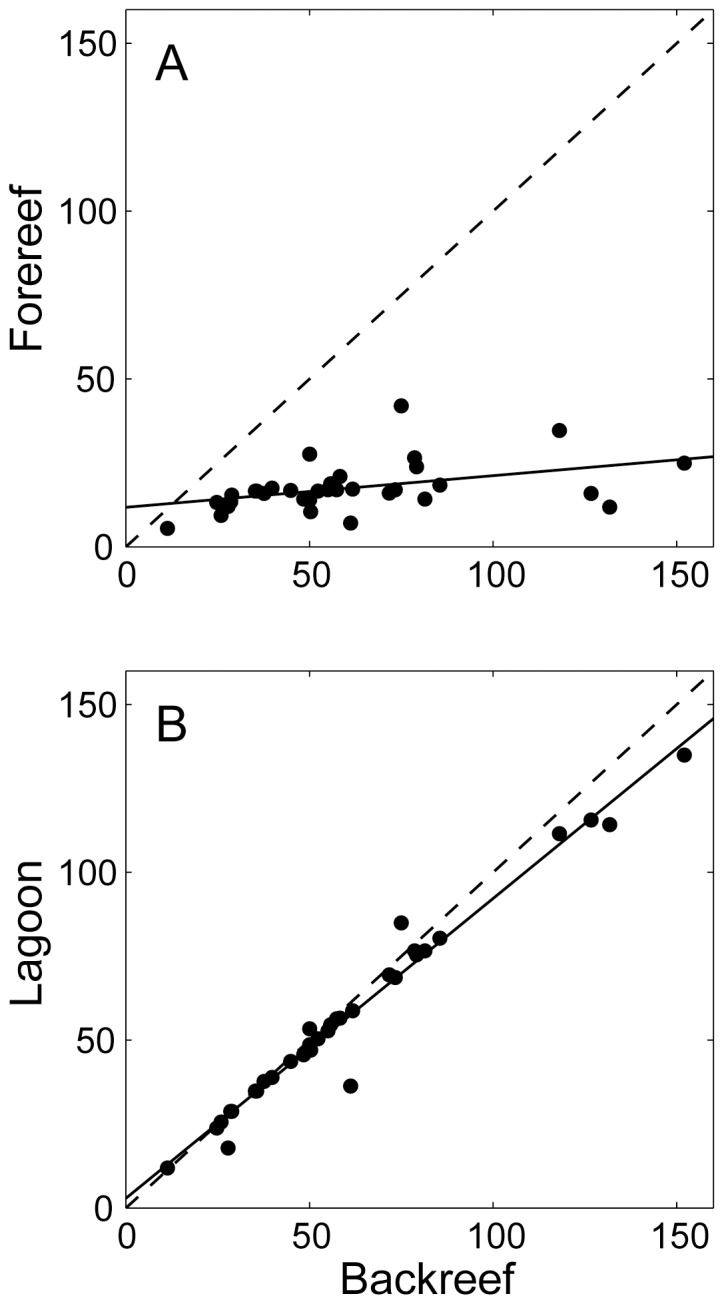
Changes in *p*CO_2_ on the forereef and lagoon versus the backreef. Amplitude of diurnal *p*CO_2_ variation 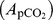 A) at the forereef and B) in the lagoon versus the backreef. The solid lines represent best-fit linear regressions in the form of A) *y* = 0.09*x*+12, *r* ^2^ = 0.18 and B) *y* = 0.89*x*+2.9, *r* ^2^ = 0.97 where *n* = 33 for both plots. The dashed lines represent 1∶1 relationships.

Diurnal changes in *p*CO_2_ across the lagoon were roughly equal in magnitude to and highly correlated with changes on the backreef, whereas diurnal changes in *p*CO_2_ across the forereef were far more modest in comparison and only weakly correlated with diurnal changes on the backreef (Fig. 8 a,b). This result was not entirely unexpected given the much higher areal rates of benthic metabolism on the reef flat versus in the lagoon (Fig. 3d). However, when we elevated rates of benthic metabolism inside the lagoon (e.g., *P*_lag_ = 330 vs. 80 mmol C m^−2^ d^−1^) as well as decreased the depth of the lagoon by one-half (*h*_c_ = 3 vs. 6 m) to simulate the high level of metabolic activity observed in some shallow lagoons [39],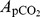 in the lagoon was still just 13% higher than at the backreef (85 vs. 75 µatm). Although 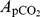 on the forereef more than doubled in this simulation relative to the model central case (42 vs. 16 µatm) due to the mixing and advection of more altered channel outflow waters, the increase in 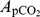 between the forereef and backreef was still more than three times greater than the increase in 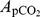 between the backreef and lagoon (+33 vs. +10 µatm). Thus, our results indicate that most of the biologically driven changes in water carbonate chemistry occurred during the relatively short transit across the reef flat (20 minutes to 3 hours, median = 50 min); and that lagoon metabolism is a generally far less important source of variation. This preliminary conclusion allows us to focus our remaining analysis primarily on the physical and biogeochemical factors driving variation in *p*CO_2_ across the reef flat itself; specifically those identified in Eq. 7 and Eq. 8.

### Predicted functional relationships

To calculate the ratio of metabolic forcing to wave forcing, it is first necessary to establish a representative benthic flux on the reef flat 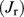 with which to calculate 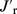 (Eq. 8). For example, changes in DIC are driven by both net production and net calcification (Eq. 14); therefore, diurnal variation in ΔDIC 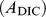 should scale according to the full diurnal range in benthic DIC fluxes. This means that benthic DIC fluxes on the reef flat should vary from a minimum equal to *−r* to a maximum equal to 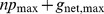, or a range equal to 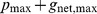. Given the complex behavior of diurnal species curves (Fig. 5) and given that we are interested in calculating the standard deviation in DIC over the course of the day (Eq. 15), it is more sensible to define a flux scale that is based on the daily integrated rates of benthic metabolism rather than short-lived maximum hourly rates. Furthermore, basing our calculations on daily integrated rates would allow us to make better use of data already reported in the literature (Table 2). Therefore, we defined the following diurnal variation flux for DIC:
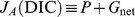
(17)For the average difference in DIC between reef and offshore waters over a 24-hour period 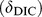, the relevant flux is the net sum of respiration minus production and net calcification:
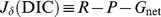
(18)such that positive values of 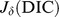 increase DIC, and vice-versa. Following similar logic, we defined the relevant fluxes for both 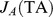 and 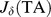 to be 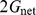 and -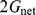, respectively. Defining equivalent benthic flux scales for dissolved CO_2_ requires accounting for the fact that calcification releases less than one mol of CO_2_ for each mol of CaCO_3_ precipitated [65]–[67]. Thus, we defined the following benthic fluxes for changes in CO_2_

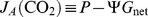
(19)

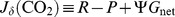
(20)where Ψ is the ratio of moles of CO_2_ released per mol of CaCO_3_ precipitated and equal to ∼0.6 for the representative tropical seawater used here (S = 35, T = 25°C, average *p*CO_2_ = 390 µatm); however, Ψ can vary between approximately 0.5 and 0.7 in tropical reef systems depending on temperature, salinity, and *p*CO_2_ [66], [68]. Finally, because we are keeping temperature and salinity constant within the domain and across all simulations, changes in water column *p*CO_2_ are in constant proportion to changes in dissolved CO_2_ gas concentration 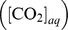 through a fixed Henry's Law constant 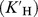


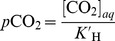
(21)Thus, for the present study we used Eq. 19 and Eq. 20 to define the benthic fluxes relevant to 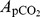 and 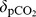, respectively, as well. The individual dependency of 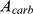 and 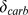 for TA, DIC, *p*CO_2_, pH, or Ω_ar_ on the ratio of metabolic to wave forcing, reef flat dimensions, and system frictional resistance is provided in Tables S1 through S5.

**Figure 9:**
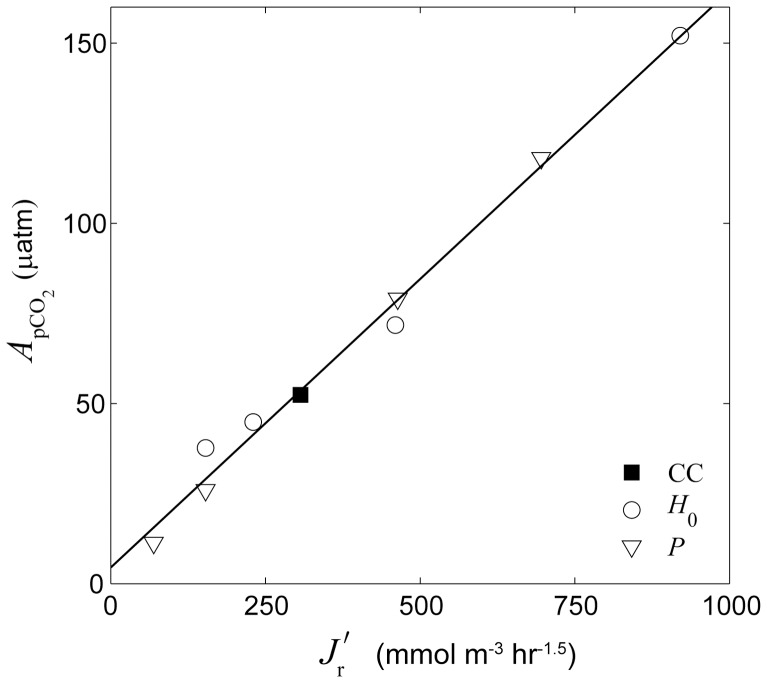
Effect of the ratio of metabolic forcing to wave forcing. Amplitude of diurnal *p*CO_2_ variation 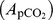 at the backreef versus 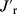 assuming 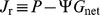 (see Eq. 19). All other variables not listed in the legend were equal to those of the central case (‘CC’) except for *G*_net_ which was set equal to 20% of *P* in all simulations shown. The solid line represents the best-fit linear regression in the form of *y* = 0.16*x*+4.5, *r* ^2^ = 0.99, *n* = 9.

**Figure 10:**
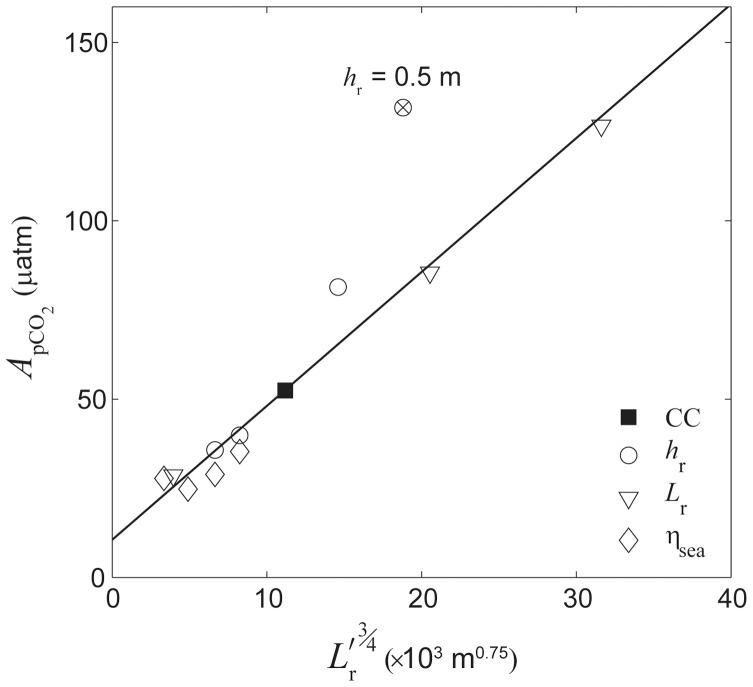
Effect of reef flat geometry. Amplitude of diurnal *p*CO_2_ variation 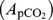 at the backreef versus 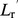 based on simulations varying reef flat depth, reef flat width, and sea level. All other variables not listed in the legend were equal to those of the central case (‘CC’). The solid line represents the best-fit linear regression in the form of *y* = 3.8*x*+10.6, *r* ^2^ = 0.96, *n* = 11. The circle with the cross represents the shallowest reef flat simulation (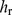 = 0.5 m) and was not included in the regression.

**Figure 11:**
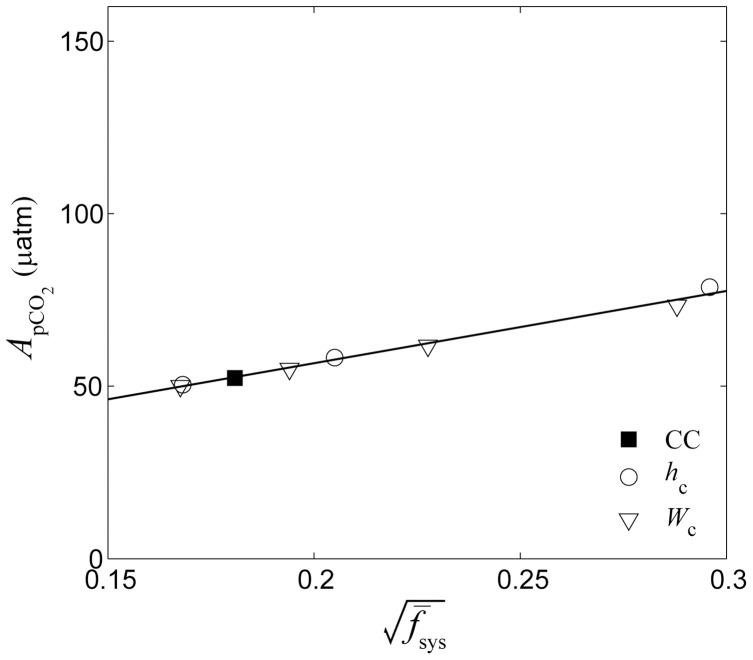
Effect of total system resistance. Amplitude of diurnal *p*CO_2_ variation 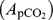 at the backreef versus 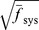 based on simulations varying channel depth and width. All other variables not listed in the legend were equal to those of the central case (‘CC’). The solid line represents the best-fit linear regression in the form of *y* = 210*x*+15, *r* ^2^ = 0.99, *n* = 8.

Changes in water column carbonate chemistry simulated by the three-dimensional model were in close agreement with predictions made by the analytical one-dimensional model. 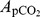 at the backreef transect increased linearly with 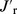 in a manner consistent with that predicted by Eq. 8 (Fig. 9). Thus, 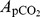 increased with both increasing rates of reef metabolism and decreasing offshore wave heights. Reducing offshore wave heights by a factor of three (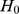 = 0.5 vs. 1.5 m) had a roughly equivalent impact on backreef 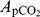
as tripling rates of benthic production and net calcification (Fig. 9). Backreef 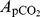 also increased linearly with 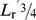 in a manner consistent with that predicted by Eq. 8 for almost all simulations (Fig. 10). Thus, changes in *p*CO_2_ were highly dependent on the depth and width of the reef flat. However, backreef 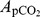 in the shallowest reef flat simulation (*h*_r_ = 0.5) deviated positively from the relationship defined by other simulations where 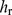, 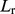 or 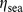 were allowed to vary and all other variables were kept constant. Increasing the offshore sea level 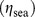 by just +0.5 m caused a ∼30% reduction in 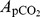 at the backreef; however, further increasing sea level from +0.5 to +4 m had little additional effect on backreef 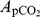. This indicated a limited sensitivity of water column carbonate chemistry to rising sea level or tide if rates of benthic metabolism remain unchanged (Fig. 10). Finally, 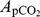 at the backreef also increased linearly with 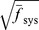 in a manner consistent with that predicted by Eq. 8; however, the dependency of 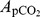 on 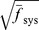 was far weaker than on 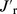 or 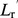 (Fig. 11).

Increasing the rate of community net calcification relative to gross primary production had only a modest effect on diurnal variations in water column *p*CO_2_. For example, changing the ratio of *G*_net_∶*P* by ±100% (i.e., changing *G*_net_ from 20% to either 0% or 40% of *P*) caused 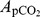
at the backreef to change by just ±8% (52±4 µatm),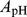 to change by just ±16% (0.047±0.007) and 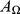 to change by just ±26% (0.27±0.07). This is because organic carbon metabolism (i.e., production and respiration) is the dominant driver of diurnal variation in water column *p*CO_2_, pH, and Ω in most coral reef systems. However, net calcification does appear to play an influential role in driving the net offset 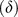 of *p*CO_2_, pH, and Ω over time scales of a day or more. Results from the central case model indicated that the uptake of CO_2_ by net production appear to balance the release of CO_2_ by net calcification over a 24-hour period such that 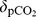 at the backreef was just −1 µatm. However, increasing the ratio of *G*_net_∶*P* to 40% increased the 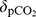 at the backreef by +14 µatm indicating that net calcification acting as a CO_2_ source was over-riding the ability of reef flat net autotrophy to act as a CO_2_ sink (Eq. 20). Not surprisingly, decreasing *G*_net_∶*P* to 0% had the opposite effect of decreasing 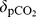 at the backreef by −15 µatm. Similarly, increasing the ratio of *G*_net_∶*P* to 40% decreased 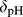 and 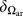 at the backreef by 0.02 and 0.17, respectively; whereas decreasing *G*_net_∶*P* to 0% increased 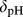 and 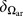 by the similar amounts. Nonetheless, the absolute magnitude of 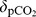, 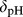, and 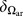 in backreef waters were always less than 20 µatm, 0.02, and 0.18; respectively, across all simulations.

## Discussion

**Figure 12:**
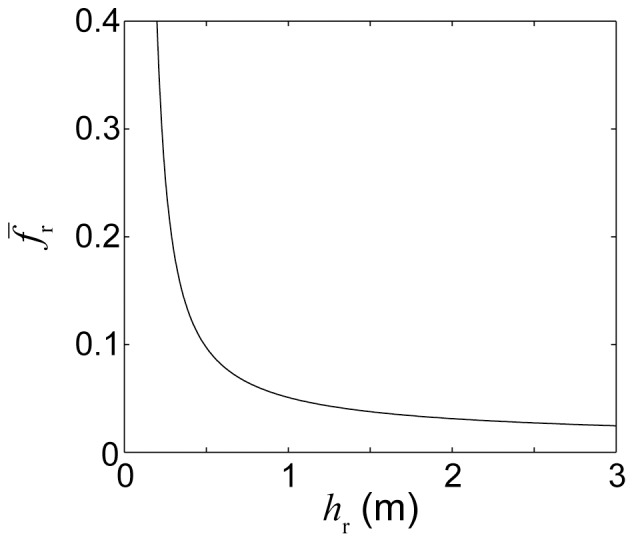
Depth-dependence of bottom friction coefficient. Reef flat bottom friction coefficient versus the average depth of the reef flat.

The minor deviation of 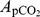 from a linear dependency on 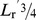 predicted by Eq. 8 for one very shallow simulation (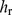 = 0.5 m, see Fig. 10) was largely the result of the frictional resistance of the reef flat increasing dramatically as the depth of the reef flat became less than ∼1 m (see Fig. 12). This behavior is consistent with prior studies of the response of mean current drag over reef communities to decreasing depth [69]–[71]. The large increase in the frictional resistance of the reef flat, in turn, caused a substantial increase in the frictional resistance of the reef system as a whole 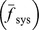. This further reduced the cross-reef transport which, in turn, increased the contact time of the water with the shallow reef flat leading to greater-than-expected alteration in water chemistry. Otherwise, the dependency of 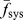 on *h*_r_ is generally weak when 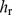 is greater than ∼0.7 m, such that the influence of *h*_r_ on changes in water chemistry is evident mainly through its influence on the reef flat length scale alone 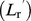. Nonetheless, the effect of decreasing *h*_r_ on changes in water chemistry will still be captured by Eq. 7, regardless of whether it acts through 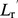 or 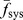.

**Figure 13:**
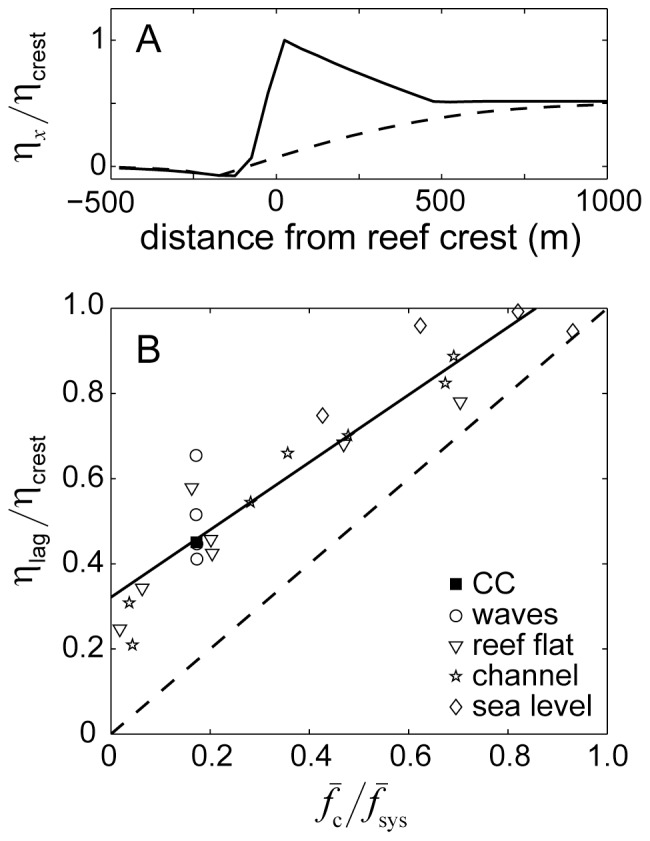
Ratio of wave setup versus ratio of system friction. A) profiles of normalized wave-driven setup across the reef flat (solid line) and channel (dashed line). B) The ratio of lagoon to reef-crest setup versus the ratio of the channel friction coefficient to the total system friction coefficient for simulations testing variation in offshore wave heights, reef flat geometry, channel geometry, and sea level. The solid line represents best-fit regression of the form of *y* = 0.8*x*+0.32 (*r* ^2^ = 0.87, *n* = 23). The dashed line represents a 1∶1 relationship.

The particularly weak dependency of 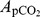 on 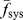 was not expected (Fig. 11); however, there are several explanations for this behavior. First, the dependency of 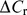 on 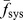 given in Eq. 7 is sub-linear 
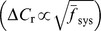
 meaning that a three-fold increase in 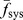 should a priori cause a less than two-fold increase in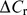. Second, Eq. 3 and Eq. 7 implicitly assume that 1) the gradient in setup driving flow through the channel extends over a distance equal to the length of the reef flat, and 2) that the setup at the entrance of the channel is ∼0 or equal to the offshore sea level. Our simulations combined with prior studies indicate that cross-reef gradients in setup through the channel are generally weaker and/or more gradual than on the reef flat; extending from beyond the reef crest to well inside the lagoon (Fig. 13a, [40]). This is the result of the inherently two-dimensional structure of both flow and setup adjacent to the channels. Flow converges on the lagoon-side of the channel and then increases seaward as along-reef inflows draining off the adjoining reefs add to the outflow. This acceleration of the channel flow is then partly resisted by setup at the channel entrance due to a gradual decline in landward wave radiation stress (Fig. 3d). Consequently, the actual setup inside the lagoon has to be greater than that predicted by the one-dimensional analytical model derived in Eq. 3 in order to generate the outflow necessary to balance the corresponding inflow across the reef flat. This means that the ratio of setup (or gravitational potential) in the lagoon to setup at the reef crest is higher than would be predicted by the corresponding ratio of channel to system resistance (Fig. 13b). This non-linear, two-dimensional effect dampens the dynamic response of 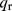 and 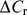 to changes in the relative width and depth of the channel further limiting the influence of 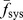 on *q*_r_ and 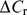. Fortunately, the relationship between 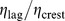 and 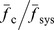 is still very linear (Fig. 13b) such that the dependency of 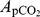 on 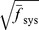 is nonetheless highly linear overall; albeit weaker than expected (Fig. 11).

**Figure 14:**
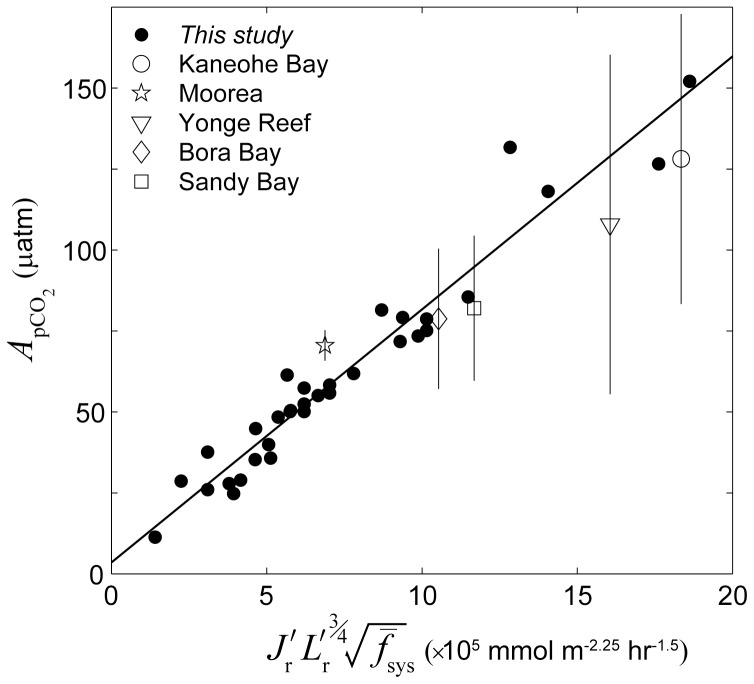
Combined effect of all factors on diurnal *p*CO_2_ variations. Amplitude of diurnal *p*CO_2_ variation 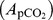 of back reef waters versus the combined influence of wave forcing, sea level, metabolism, reef flat geometry, and system frictional resistance for all 33 simulations (dark circles, see Eq. 7 and Eq. 8). For all data shown, 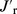 was calculated assuming 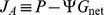 (see Eq. 19). The solid line represents a best-fit linear regression of the simulations in the form of *y* = 7.8*x*+3.5 (MSE = 8 µatm, *r* ^2^ = 0.94, *n* = 33). Also shown are the average diurnal *p*CO_2_ variation amplitudes observed in real reef systems [24], [25], [56], [57] normalized to a temperature of 25°C (see Eq. 21) for which there was enough supporting hydrodynamic and biogeochemical data (see Tables 2 and 3, the vertical lines represent ±1 std. dev.). The best-fit regression for the observed mean 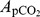 versus the *x*-axis term is *y* = 5*x*+29 (MSE = 7 µatm, *r* ^2^ = 0.94, *p*<0.005, *n* = 5, not shown) and is not significantly different from the regression derived from the model simulations (*p*>0.25).

**Figure 15:**
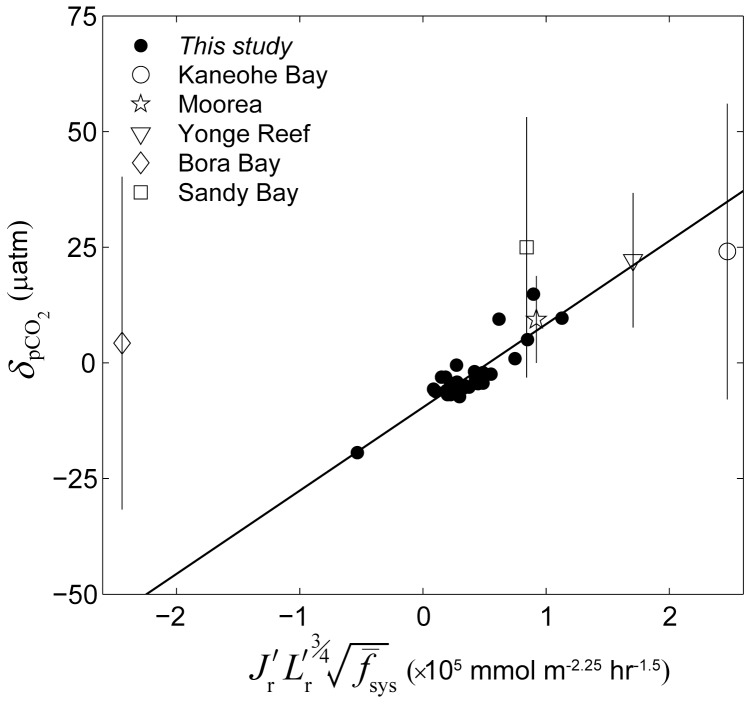
Combined effect of all factors on the net offset in *p*CO_2_. Difference between the *p*CO_2_ of backreef waters and offshore waters averaged over a 24-hour period 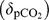 versus the combined influence of wave forcing, sea level, metabolism, reef flat geometry, and system frictional resistance for all 33 simulations (dark circles, see Eq. 7 and Eq. 8). For all data shown, 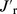 was calculated assuming 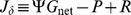 (see Eq. 20). The solid line represents a best-fit linear regression of the simulations in the form of *y* = 18*x*–9.6 (MSE = 3 µatm, *r* ^2^ = 0.76, *n* = 33). Also shown are the average offsets in the *p*CO_2_ of reef waters relative to offshore waters observed in real reef systems [24], [25], [56], [57] normalized to a temperature of 25°C (see Eq. 21) for which there was enough supporting hydrodynamic and biogeochemical data (see Tables 2 and 3; the vertical lines represent ±1 std. dev.). The best-fit regression for the observed 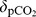 versus the *x*-axis term is *y* = 4*x*+14 (MSE = 7 µatm, *r* ^2^ = 0.64, *p*<0.06, *n* = 5, not shown).

Taken in total, the combined dependency of 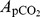 on 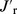, 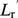, and 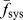 produced by the three-dimensional model was very consistent with that predicted by the terms shown in Eq. 7 and Eq. 8 derived in our analytical one-dimensional model for all 33 simulations (*r* ^2^ = 0.94, Fig. 14). Although we developed our numerical model around a flexible idealized reef, average
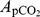
observed in real reef systems showed very good agreement with predictions made by our analytical one-dimensional model as well (*r* ^2^ = 0.94, Fig. 14). The combined dependency of 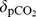
on 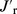, 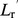, and 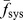 produced by the three-dimensional model was also very consistent with that predicted by the one-dimensional model assuming the appropriate benthic flux scale for 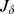 (*r* ^2^ = 0.76, Fig. 15). There was also reasonably good agreement between average observed 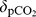
on real reefs and predictions made by our analytical one-dimensional model (*r* ^2^ = 0.64, Fig. 15); however, the slope of the regression of observed 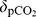
versus combined predictive factors was less than that for the idealized simulations (4×10^−5^ vs. 18×10^−5^ µatm m^2.25^ hr^1.5^ mmol^−1^, *p*<0.05). This is largely due to the influence of the data from Bora Bay exhibiting 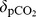 which clustered around 5±30 µatm despite substantial net CO_2_ uptake measured in that reef system (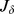
<0). One reason for the larger discrepancy between predicted 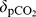 versus simulated and observed 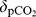 in comparison to with 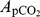 is that 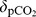 was considerably smaller than 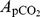
for both our idealized simulations and for real reefs. In our simulations 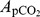 was ∼15 times higher than 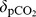
on average while for the real reef data 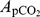 was ∼5 times higher than 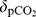
on average. Such a high signal-to-offset ratio would make the estimates of 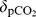 from either model simulations or in situ observations far more prone to uncertainty than estimates of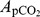. This is likely because the release of CO_2_ in most real reefs from calcification and respiration is largely offset by the uptake of CO_2_ from photosynthetic production (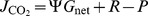, [67]) such that most reefs are only modest sources of CO_2_ [67], [72], [73]. The sum of daily integrated CO_2_ fluxes for the central model case equaled (0.6×130)–660+610 or just +30 mmol CO_2_ m^−2^ d^−1^; a value consistent with prior estimates made for coral reefs worldwide (+10 to +30 mmol CO_2_ m^−2^ d^−1^ [68]). Not only is this CO_2_ flux much less than the error in most estimates of either *P*, *R*, or *G*_net_ alone [24], [26], [45], it ultimately produced 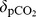
of just −1 to −2 µatm in backreef and lagoon waters given typical offshore wave heights, reef dimensions, and rates of benthic metabolism.

Tides can provide another source of variation in 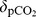 for real reefs that were not explicitly considered in the present simulations. Most tidal modes occur on periods that are not perfectly in phase with the 24-hour diurnal cycle causing the minimum and maximum tidal elevations to occur at different times of day. This, in turn, causes continually varying phase differences between the lows and highs of a strongly diurnal tide and the maximum and minimum rates of benthic metabolism [33]. Consequently, 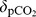 will be much more negative on days when low tides occur at mid-day and much more positive on days when low tides occur at night (a similar logic can be applied to the other carbonate species). Where tidal variations are large (>2 m), such phase-driven deviations in carbonate chemistry can be quite large [30]; especially given the synergistic effects of very shallow reef flats on amplifying the metabolic signal as well as decreasing cross-reef transport through increased frictional resistance (Fig. 10). We did not pursue such tidal-interaction simulations given the computational expense of running all of the simulations over a full lunar cycle (∼1 month) and given prior results showing that differences in tidal phase affected changes in carbonate chemistry mainly through their influence on overall mean sea level [33] (the latter being captured in our simulations). Regardless, these phase-driven positive and negative deviations in 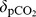 should offset one another when averaged over several weeks or more resulting in long-term 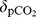 that approach values similar to those we report here. The present model may not apply, however, to extreme low tide conditions when offshore sea level plus wave-driven setup is below the level of the reef crest and wave-driven flow may cease to occur.

Our results indicate that many wave-driven reef systems will be unable to sustain dramatic net long-term changes in their carbonate chemistry relative to offshore waters given typical rates of benthic metabolism, reef morphology, wave forcing, and frictional resistance. There are examples of reef systems with seasonally higher backreef or lagoon 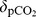 (>50 µatm [23], [25], [30]), but these higher values are probably as dependent on the physical characteristics of the specific reef system (e.g., wave forcing, morphology, and sea level) as they are on the summary effects of production, respiration, and calcification on net CO_2_ fluxes. Furthermore, in the present study we defined 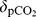 relative to offshore waters that were in equilibrium with the atmosphere. It is not uncommon, however, for the carbonate chemistry of offshore waters to be in disequilibrium with the atmosphere due to oceanic processes such as upwelling, net pelagic production, and those affecting the gas solubility of surface waters [23], [24], [74], [75]. Such larger-scale ocean processes may explain why long-term differences between the *p*CO_2_ of reef waters and that of the atmosphere are often much greater than long-term differences between the *p*CO_2_ of reef waters and offshore waters (i.e., 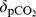), especially on seasonal time scales [23]. This is well beyond the scope of the present study, but should be considered in future investigations.

### Larger reef systems and atolls

**Figure 16:**
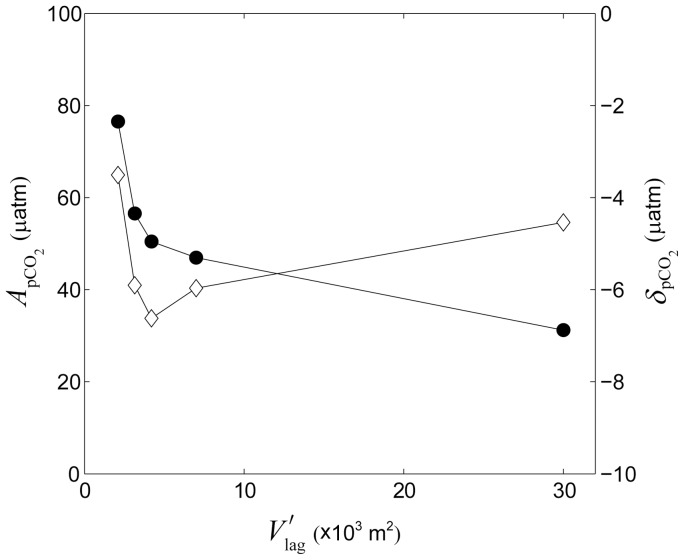
Changes in *p*CO_2_ versus lagoon volume. Amplitude of diurnal *p*CO_2_ variation (closed circles, left *y*-axis) and net offset in the *p*CO_2_ of lagoon waters relative to offshore waters over a 24-hour period (open diamonds, right *y*-axis) versus lagoon volume per width of reef flat 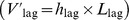.

Our simulations focused mainly on coastally bound reef-lagoon systems with modest lagoon sizes (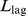
≤1500 m) and residence times that ranged from several hours to just over a day. This raises the question of whether variations in lagoon carbonate chemistry differ for much larger reef systems with lagoons that are tens of kilometers in size (e.g., New Caledonia; Kwajalein and Majuro in the Marhshall Islands; Chuuk and Ulithi in Micronesia; Rangiroa, Tikehau, Muraroa and Ahe in French Polynesia; Huvadhoo and Ari in the Maldives; Glovers Reef and Lighthouse Reef in Belize; Bermuda in the west Atlantic; etc.) and whose residence times vary from weeks to months [76]–[79]. Our results, however, indicated that most of the changes in carbonate chemistry occurred over the reef flat and that changes in carbonate chemistry within the lagoon decreased with increasing lagoon volume (Fig. 16). Interestingly, the size of reef flats tend to remain relatively constrained (generally 200 to 1000 m, [80]) even though the size of reef lagoons range by roughly two orders of magnitude (e.g., compare ∼50 km for Chuuk, Micronesia versus 0.5 km for Sandy Bay, Ningaloo Reef, Australia). Given the well-defined range of benthic reef flat metabolic rates as well (Table 2), this implies that the submerged reef rims of large atolls should be of roughly the same size and reactivity as those found on nearshore fringing reef-lagoon systems. Furthermore, previous work has shown that the *P*∶*R* ratio for entire reef systems are even closer to unity than for individual reef zones; thus, indicating that net CO_2_ fluxes decrease when integrating over increasingly larger spatial scales [39]. In fact, both prior and ongoing observations have shown that net offsets in the *p*CO_2_ of lagoon waters relative to offshore were less than 30 µatm on average inside Majuro atoll (10 km×40 km, 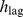 = 35 m, [81]), less than 30 µatm inside the Great Barrier Reef (∼50 km×∼2,000 km, 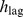 = 35 m, [82]), and generally less than 50 µatm inside Chuuk atoll (50 km×50 km, 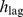 = 50 m, http://www.pmel.noaa.gov/co2). These changes are modest in comparison with the hundreds of µatm increase in *p*CO_2_ anticipated to occur over the 21^st^ century.

Given that net ecosystem production becomes smaller with increasing spatial and temporal scales, long-term changes in the carbonate chemistry of deep lagoons should be driven more by the release of TA from the dissolution of lagoon sediments:
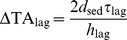
(22)where 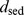 is the rate of sediment carbonate mineral dissolution and 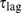 is the residence time of lagoon waters with respect to surrounding ocean. Rates of dissolution in carbonate sediments of reef systems range between 0.0004 to 0.8 mmol m^−2^ hr^−1^ with a median value of ∼0.3 mmol m^−2^ hr^−1^ or ∼7 mmol m^−2^ d^−1^ [83]. Assuming a 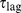 of 1 to 2 months [77]–[79] and an average lagoon depth of 46 m [84] for larger atolls, then these lagoons should exhibit long-term increases in TA relative to waters flowing off the reef flat on the order of just ∼10 µeq kg^−1^. This suggests that variations in carbonate chemistry within such deep lagoons could be more influenced by planktonic growth and metabolism [85]. We caution that reef systems enclosing large but shallow lagoons with long residence times (>weeks) and/or populated by a high density of shallow patch reefs (e.g., Bermuda or Glovers Reef, Belize) could exhibit even greater longer-term shifts in carbonate chemistry relative to offshore waters. Regardless, any additional long-term offsets in lagoon water column carbonate chemistry could be roughly estimated by applying the appropriate variation of Eq. 22 using spatially averaged rates of benthic net production or net calcification [86], [87], and adding them to net changes in the carbonate chemistry of waters exported from the reef flat as predicted by the present model 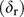.

## Conclusions

The main objective of the present study was to demonstrate and to better understand how spatial and temporal variation in water column carbonate chemistry are influenced by the different physical and biogeochemical attributes of a reef system acting in combination, not just by rates of benthic metabolism alone. Our results combined with data in the literature allow us to conclude the following: Firstly, most of the changes in carbonate chemistry occur during the transit of water across the reef flat, although these changes can be further augmented by particularly high rates of metabolism in shallow lagoons. Secondly, changes in carbonate chemistry are as sensitive to the combined length and depth of the reef flat as they are on the ratio of metabolic to wave forcing; however, they are much less sensitive to variations in channel morphology and to the overall frictional resistance of the reef system. Thirdly, the long-term (weeks to months) net offset in water column *p*CO_2_ relative to offshore waters for many reef systems is likely to be marginal due to 1) the release of CO_2_ from net community calcification being mostly offset by the uptake of CO_2_ from net community production, and 2) physically constrained limits on wave-driven circulation, the length and depth of the reef flat, and the overall frictional resistance of the entire reef system. More importantly, our results demonstrate that it is not possible to properly interpret observed spatial and temporal variations in water column carbonate chemistry, nor to determine the significance of these observations with respect to reef systems in general, without adequate hydrodynamic and morphological data. We suggest that future field studies and monitoring programs aimed at tracking the in situ impacts of ocean acidification on coral reef systems collect such physical information in addition to detailed measurements of water column chemistry. The analytical model we present here thus provides an additional tool to advance these efforts in wave-driven coral reef systems worldwide.

## Supporting Information

Appendix S1 **Derivation of wave setup formulation.**
(DOC)

Appendix S2 **Derivation of cross-reef transport formulation.**
(DOC)

Appendix S3 **Bottom current drag formulations.**
(DOC)

Appendix S4 **Net production and net calcification parameterizations.**
(DOC)

Movie S1 **Time-dependent changes in the depth-averaged Total Alkalinity (TA) of reef waters relative to offshore waters over a 24-hour period.**
(WMV)

Movie S2 **Time-dependent changes in the depth-averaged Dissolved Inorganic Carbon (DIC) of reef waters relative to offshore waters over a 24-hour period.**
(WMV)

Movie S3 **Time-dependent changes in the depth-averaged** ***p*** **CO_2_ of reef waters relative to offshore waters over a 24-hour period.**
(WMV)

Movie S4 **Time-dependent changes in the depth-averaged pH of reef waters relative to offshore waters over a 24-hour period.**
(WMV)

Movie S5 **Time-dependent changes in the depth-averaged aragonite saturation state of reef waters relative to offshore waters over a 24-hour period.**
(WMV)

Table S1 **Variation amplitude and time-average difference in depth-averaged Total Alkalinity (TA) between reef waters and offshore waters over a 24-hour period.**
(DOC)

Table S2 **Variation amplitude and time-average difference in depth-averaged Dissolved Inorganic Carbon (DIC) between reef waters and offshore waters over a 24-hour period.**
(DOC)

Table S3 **Variation amplitude and time-average difference in depth-averaged** ***p*** **CO_2_ between reef waters and offshore waters over a 24-hour period.**
(DOC)

Table S4 **Variation amplitude and time-average difference in depth-averaged pH between reef waters and offshore waters over a 24-hour period.**
(DOC)

Table S5 **Variation amplitude and time-average difference in depth-averaged aragonite saturation state between reef waters and offshore waters over a 24-hour period.**
(DOC)
