# Supplementary material for: Physical and Biological Controls on the Carbonate Chemistry of Coral Reef Waters: Effects of Metabolism, Wave Forcing, Sea Level, and Geomorphology
Source: PLoS One. 2013 Jan 9;8(1):e53303. doi: 10.1371/journal.pone.0053303 (PMC3541250; doi:10.1371/journal.pone.0053303)
Supplement: Appendix S1 — Derivation of wave setup formulation. (DOC) [file pone.0053303.s001.doc]

### Appendix S1: Wave setup

Gourlay and Colleter 2005 [1] developed an analytical formulation to describe wave-driven setup $\left(\eta_{\text{crest}}\right)$ at the reef crest as a function of incident wave power (proportional to $H_{\text{o}}^{2}T$) which can be approximated as

$$\eta_{\text{crest}} = \frac{3K_{\text{p}}\sqrt{g}}{64\pi}\cdot\frac{H_{0}^{2}T}{\left(d_{\text{r}}^{\frac{3}{2}} +3K_{\text{p}}q_{\text{r}}/2g^{\frac{1}{2}}\right)}P_{\text{T}}$$

where $K_{\text{p}}$ is the empirical reef profile factor, $H_{0}$ is the offshore wave height, *T* is the wave period, $d_{\text{r}}$ is the total depth of the reef flat equal to the sum of the reef flat depth $\left(h_{\text{r}}\right)$and the reef flat setup $\left(d_{\text{r}} = h_{\text{r}} + \eta\right)$, $q_{\text{r}}$ is the cross-reef transport per width of reef, and *g* is the rate of gravitational acceleration. $P_{\text{T}}$ is the wave power transmission parameter defined as

$$P_{\text{T}} = 1 - 4\pi\gamma_{\text{r}}^{2}\frac{d_{\text{r}}^{\frac{5}{2}}}{H_{0}^{2}Tg^{\frac{1}{2}}} - 16\pi\frac{q_{r}^{3}}{g^{2}d_{r}^{2}H_{\text{o}}^{2}T}$$

assuming that wave reflection off the reef front is negligible and $\gamma_{r}$ is the maximum height of waves relative to depth on the reef flat. Gourlay and Colleter 2005 found that $K_{\text{p}}$ = 0.8 for steep-faced reefs, however, this value can be lower for reefs with more gentle forereef slopes [2]. Measurements across many reefs reported in the literature indicate that for most reef $H_{0}$ is ~1.5 m, $h_{\text{r}}$ is ~1.5 m and $q_{\text{r}}$are ~0.2 m^-2^ s (Table 1). Assuming these characteristics and a wave period of ~10 s (a value that is intermediate to locally generated wind waves 5 to 8 s and oceanic swell 12 to 16 s), we can simplify Eq. 1 to

$$\eta_{\text{crest}} = \frac{3K_{\text{p}}\sqrt{g}}{64\pi}\cdot\frac{H_{0}^{2}T}{d_{\text{r}}^{\frac{3}{2}}}P_{\text{T}}$$

while introducing an error of only less than ~1 cm. Similarly, it is easy to show that $P_{\text{T}}$ can be simplified to

$$P_{\text{T}} = 1 - 4\pi\gamma_{\text{r}}^{2}\frac{d_{\text{r}}^{\frac{5}{2}}}{H_{0}^{2}Tg^{\frac{1}{2}}}$$

while introducing less than 1% error. In fact, for the typical reef conditions listed above, $P_{\text{T}}$ = 0.97 or ≈ 1. Thus, given typical reef morphology and wave conditions, $\eta_{\text{crest}}$can be estimated by the following with an error of just ~1 cm

$$\eta_{\text{crest}} = \frac{3K_{\text{p}}\sqrt{g}}{64\pi}\cdot\frac{H_{0}^{2}T}{d_{\text{r}}^{\frac{3}{2}}}$$

1. Gourlay MR, Colleter G (2005) Wave-generated flow on coral reefs-an analysis for two-dimensional horizontal reef-tops with steep faces. Coastal Eng 52: 353-387.

2. Lowe RJ, Falter JL, Monismith SG, Atkinson MJ (2009) Numerical model of wave transformation and circulation within a barrier reef-lagoon system: Kaneohe Bay, Hawaii. J Geophys Res 114.
