# Supplementary material for: Physical and Biological Controls on the Carbonate Chemistry of Coral Reef Waters: Effects of Metabolism, Wave Forcing, Sea Level, and Geomorphology
Source: PLoS One. 2013 Jan 9;8(1):e53303. doi: 10.1371/journal.pone.0053303 (PMC3541250; doi:10.1371/journal.pone.0053303)
Supplement: Appendix S2 — Derivation of cross-reef transport formulation. (DOC) [file pone.0053303.s002.doc]

### Appendix S2: Cross-reef transport

Assuming that wave dissipation across the shallow reef flat is minimal, the loss in setup across the reef flat and channel is balanced by bottom friction which can be expressed as a function of the bottom friction coefficient $\left(\bar{f}_{D}\right)$, the total depth, and the depth-integrated transport according to Lowe et al. 2009 [1]

$$\frac{\Delta\eta}{\Delta x} = -\frac{\bar{f}_{D}q\left|q\right|}{2gd^{3}}$$

For the reef flat and channel, we can define Δ*x* to be equal to $L_{\text{r}}$, or the length of the reef flat in the cross-reef direction. Combining Eq. 5 and Eq. 6 and assuming that $d_{\text{r}} \approx h_{\text{r}}$, and $d_{\text{c}} \approx h_{\text{c}}$we get the following relationship

$$\eta_{\text{crest}} = \frac{\bar{f}_{\text{r}}q_{\text{r}}^{2}}{2gh_{\text{r}}^{3}}L_{\text{r}} + \frac{\bar{f}_{\text{c}}q_{\text{c}}^{2}}{2gh_{\text{c}}^{3}}L_{\text{r}}$$

Continuity requires that the total inflow across the reef flat be equal to the total outflow through the channel

$$q_{\text{c}}W_{\text{c}} = q_{\text{r}}W_{\text{r}}$$

Where $W_{\text{r}}$ and $W_{\text{c}}$ are the widths of the reef and channel, respectively, in the alongshore direction. Combining Eq. 3, Eq. 7, and Eq. 8 and re-arranging results in the following relationship between cross-reef transport and incident wave forcing

$$q_{\text{r}} = K'_{\text{p}}\left(\frac{H_{\text{o}}^{2}T}{h_{\text{r}}^{\frac{3}{2}}L_{\text{r}}}\right)^{\frac{1}{2}}\left(\frac{\bar{f}_{\text{r}}}{2h_{\text{r}}^{3}} + \frac{\bar{f}_{\text{c}}}{2h_{c}^{3}W_{c}^{*2}}\right)^{-\frac{1}{2}}$$

where $K'_{\text{p}}$ is equal to $\left(3K_{\text{p}}g^{\frac{3}{2}}/64\pi\right)^{\frac{1}{2}}$or ≈ 0.6 if $K_{\text{p}}$ = 0.8, and $W_{\text{c}}^{*}$is the ratio of the channel width to reef width $\left(W_{\text{c}}^{*} \equiv W_{\text{c}}/W_{\text{r}}\right)$ [2]. It is important to note that $\bar{f}_{c}$ was calculated accounting for wave-current interactions (see section on Bottom current drag below).

1. Lowe RJ, Falter JL, Monismith SG, Atkinson MJ (2009) Numerical model of wave transformation and circulation within a barrier reef-lagoon system: Kaneohe Bay, Hawaii. J Geophys Res 114.

2. Lowe RJ, Hart C, C.B. P (2010) Morphological constraints to wave-driven circulation in coastal reef-lagoon systems: A numerical study. J Geophys Res -Oceans 115: 13 pp.
