# Supplementary material for: Physical and Biological Controls on the Carbonate Chemistry of Coral Reef Waters: Effects of Metabolism, Wave Forcing, Sea Level, and Geomorphology
Source: PLoS One. 2013 Jan 9;8(1):e53303. doi: 10.1371/journal.pone.0053303 (PMC3541250; doi:10.1371/journal.pone.0053303)
Supplement: Appendix S3 — Bottom current drag formulations. (DOC) [file pone.0053303.s003.doc]

### Appendix S3: Bottom current drag

The depth-averaged current drag coefficient can be calculated from the depth *h* and roughness length scale $z_{0}$ according to a close approximation of the logarithmic rough-wall boundary layer [1]

$$\bar{f}_{D} = \frac{0.32}{\left(\ln\left(\frac{h}{z_{0}}\right) -1 \right)^{2}}$$

For areas such as open channels where wave heights and therefore near-bottom wave orbital motions are significant, wave enhancement of bottom friction roughness coefficients need to be accounted for [1]

$$\bar{f}_{cw} = \left(1 + 1.2\left(\frac{\tau_{w}}{\tau_{w} + \tau_{c}}\right)^{3.2}\right)\bar{f}_{c}$$

where $\bar{f}_{cw}$ is the friction coefficient in the presence of waves, $\bar{f}_{c}$ is the friction coefficient in the case of pure, steady current, $\tau_{w}$ is the maximum bottom wave stress in the case of pure wave orbital motion, and $\tau_{c}$ is the bottom stress in the case of a pure, steady current. $\tau_{c}$ is calculated according to a standard quadratic relationship

$$\tau_{c} = \frac{1}{2}\bar{f}_{c}\rho\bar{U}_{c}^{2}$$

where *ρ* is the density of seawater and $\bar{U}_{c}$ is the depth-averaged current. $\tau_{w}$ is calculated based on the maximum near-bottom wave orbital speed $\tilde{U}_{b,\max}$ and the wave friction coefficient $f_{w}$

$$\tau_{w} = \frac{1}{2}f_{w}\rho\tilde{U}_{b}^{2}$$

$\tilde{U}_{b,\max}$ can be estimated from linear wave theory according to

$$\tilde{U}_{\text{b},\max} = \frac{H\omega}{2\sinh\left(k h\right)}$$

$f_{w}$ can be calculated based on $\tilde{U}_{\text{b},\max}$ and the bottom roughness length scale according to an empirical relationship provided by Soulsby 1995 [1].

$$f_{w} = 1.39\left(\frac{\tilde{U}_{\text{b},\max}}{\omega z_{0}}\right)^{-0.52}$$

1. Soulsby RL (1995) Bed shear-stresses due to combined waves and currents. In: Stive MJF, de Vriend HJ, Fredsoe J, Hamm L, Soulsby RL et al., editors. Advances in Coastal Morphodynamics. Delft, Netherlands: Delft Hydraulics. pp. 20-23.
