# Supplementary material for: Physical and Biological Controls on the Carbonate Chemistry of Coral Reef Waters: Effects of Metabolism, Wave Forcing, Sea Level, and Geomorphology
Source: PLoS One. 2013 Jan 9;8(1):e53303. doi: 10.1371/journal.pone.0053303 (PMC3541250; doi:10.1371/journal.pone.0053303)
Supplement: Appendix S4 — Net production and net calcification parameterizations. (DOC) [file pone.0053303.s004.doc]

### Appendix S4: Net production and net calcification

Prior work has shown that hourly rates of net community production often do not saturate with light intensity [1-6] due to canopy effects [7]. Using continuous measurements of benthic metabolism, Falter et al. 2011, 2012 [5,6] found that hourly rates of gross community production in reef communities could be related to light according to a power law of the form

$$p = a_{1}E_{\text{d}}^{0.6}$$

where *E*_d_ is the downwelling planar flux of Photosynthetically Active Radiation (PAR) at the benthos and *a*_1_ is a coefficient which depends upon the rate of community production. Hourly rates of net community calcification in reef communities are generally linearly correlated with *E*_d_ [1-4,6,8]

$$g_{\text{net}} = a_{2}E_{\text{d}}$$

where *a*_2_ is a coefficient which depends upon the rate of community net calcification. While the exact functional dependency of calcification on light, temperature, net production, and/or carbonate chemistry remains the subject of ongoing study; or present objective was simply to create realistic diurnal variations in *g*_net_ (and similarly *p*) which sum to daily rates of metabolism consistent with values reported in the literature (Table 2). Thus, in all simulations we assumed the same constant temperature (25°C) and the same diurnal light cycle given as

$$\begin{matrix} E_{\text{d}}\left(t\right) = E_{\text{d},\max}\sin^{2}\left(\frac{\pi\left(t- t_{\text{sr}}\right)}{t_{\text{ss}}- t_{\text{sr}}} \right) \text{for} t_{\text{sr}}\leq t\leq t_{\text{ss}} \\ E_{\text{d}}\left(t\right) = 0 \text{otherwise} \end{matrix}$$

where *t*_sr_ and *t*_ss_ represent sunrise (6 am) and sunset (6 pm) respectively for a 12-hour day. Given the constraints of daily *P* and *G*_net_ imposed by Eq. 10 in the main text, we can thus eliminate *E*_d,max_, *a*_1_, and *a*_2_ altogether to scale diurnal changes in *p* directly on daily integrated *P*

$$\begin{matrix} p(t) = p_{\max}\sin^{1.2}\left(\frac{\pi\left(t- t_{\text{sr}}\right)}{t_{\text{ss}}- t_{\text{sr}}} \right) \text{for} t_{\text{sr}}\leq t\leq t_{ss} \\ p\left(t\right) = 0 \text{otherwise} \end{matrix}$$

where the sin^1.2^ term arises from the non-linear dependency of hourly gross production on light (Eq. 16). We can further define the maximum hourly rate of gross production based on the daily integrated gross production

$$P = p_{\max}\int_{t_{sr}}^{t_{ss}} \sin^{1.2}\left(\frac{\pi\left(t- t_{\text{sr}}\right)}{t_{\text{ss}}- t_{\text{sr}}} \right) dt$$

Integrating Eq. 20, solving for $p_{\max}$and substituting into Eq. 19 yields the following result

$$\begin{matrix} p(t) = \frac{1.68P}{t_{\text{ss}} - t_{\text{sr}} }\sin^{1.2}\left(\frac{\pi\left(t- t_{\text{sr}}\right)}{t_{\text{ss}}- t_{\text{sr}}} \right) \text{for} t_{\text{sr}}\leq t\leq t_{ss} \\ p\left(t\right) = 0 \text{otherwise} \end{matrix}$$

We can also scale diurnal changes in *g*_net_ directly on daily integrated *G*_net_ as

$$\begin{matrix} g_{\text{net}}(t) = \frac{2G_{\text{net}}}{t_{\text{ss}} - t_{\text{sr}} }\sin^{2}\left(\frac{\pi\left(t- t_{\text{sr}}\right)}{t_{\text{ss}}- t_{\text{sr}}} \right) \text{for} t_{\text{sr}}\leq t\leq t_{\text{ss}} \\ g_{\text{net}}\left(t\right) = 0 \text{otherwise} \end{matrix}$$

While there is sparse data on depth-dependent changes in reef primary producer metabolism, work by Mass et al. 2007 [9] have shown that production, respiration, and calcification in corals decrease with depth at a rate much more slowly than light; indicative of the ability of reef primary producers to adapt to the lower light levels deeper on the reef. In that study, calcification rates were constant to a depth that was equal to roughly one-half the optical depth or $h_{\text{m0}}$ = 0.5/$k_{\text{d}}$, where $k_{\text{d}}$ is the diffuse attenuation coefficient for planar downwelling PAR. Below $h_{\text{m0}}$, rates of production, respiration, and calcification decreased exponentially at a constant rate roughly proportional to ~$\frac{2}{3}$*k*_d_. Therefore, we modeled the depth-dependency of *p*, *r*, and *g*_net_ in the present study according to

$$M(h) = \left\{\begin{matrix} M & h\leq h_{\text{m}0} \\ Me^{\left(-\frac{2}{3}k_{\text{d}}(h-h_{\text{m}0})\right)} & h_{\text{m}0} <h \end{matrix}\right.$$

where *M* represents *p*, *r*, or *g*_net_ as defined in the main text. For the present study we chose $k_{\text{d}}$ = 0.15 m^-1^ based on data from our own studies and that provided in the literature yielding $h_{\text{m0}}$ = 3.3 m [6,8,10-12]. Prior modelling efforts have indicated that variations in water chemistry are only weakly dependent on the exact relationship between benthic metabolism and depth given that the chemical signature of benthic metabolism becomes naturally diluted by an increasingly deeper water column [8]. Thus, simulations results are relatively insensitive to the exact formulation used in Eq. 23.

1. Barnes DJ, Devereux MJ (1984) Productivity and calcification on a coral reef: A survey using pH and oxygen electrodes. J Exp Mar Biol Ecol 79: 213-231.

2. Gattuso J-P, Pinchon M, Delesalle B, Canon C, Frankignoulle M (1996) Carbon fluxes in coral reefs. I. Lagrangian measurement of community metabolism and resluting air-sea CO_2_ disequilibrium. Mar Ecol Prog Ser 145: 109-121.

3. Yates KK, Halley RB (2003) Measuring coral reef community metabolism using new benthic chamber technology. Coral Reefs 22: 247-255.

4. Kraines S, Suzuki Y, Omori T, Shitashima K, Kanahara S, et al. (1997) Carbonate dynamics of the coral reef system at Bora Bay, Miyako Island. Mar Ecol Prog Ser 156: 1-16.

5. Falter JL, Atkinson MJ, Schar DW, Lowe RJ, Monismith SJ (2011) Short-term coherency between gross primary production and community respiration in an algal-dominated reef flat. Coral Reefs 30: 53-58.

6. Falter JL, Lowe RJ, Atkinson MJ, Cuet P (2012) Seasonal coupling and de-coupling of calcification rates from coral reef metabolism and carbonate chemistry at Ningaloo Reef, Western Australia. J Geophys Res Oceans 117: 14 pp.

7. Binzer T, Sand-Jensen K, Middleboe A-L (2006) Community photosynthesis of aquatic macrophytes. Limnol Oceanogr 51: 2722-2733.

8. Zhang Z, Falter JL, Lowe R, G. I (2012) The combined influence of hydrodynamic forcing and calcification on the spatial distribution of alkalinity in a coral reef system. J Geophys Res 117: 18 pp.

9. Mass T, Einbinder S, Brokovich E, Shashar N, Vago R, et al. (2007) Photoacclimation of *Stylophora pistillata* to light extremes: metabolism and calcification. Mar Ecol Prog Ser 334: 93-102.

10. Michael KJ, Veal CJ, Nunez M (2012) Attenuation coefficients of ultraviolet and photosynthetically active wavelengths in the waters of Heron Reef, Great Barrier Reef, Australia. Mar Fresh Res 63: 142-149.

11. Yentsch CS, Yentsch CM, Cullen JJ, Lapointe BE, Phinney DA, et al. (2002) Sunlight and water transparency: cornerstones in coral research. J Exp Mar Biol Ecol 268: 171-183.

12. Hochberg EJ, Atkinson MJ (2008) Coral reef benthic productivity based on optical absorptance and light-use efficiency. Coral Reefs 27: 49-59.
