# Supplementary material for: Physical and Biological Controls on the Carbonate Chemistry of Coral Reef Waters: Effects of Metabolism, Wave Forcing, Sea Level, and Geomorphology
Source: PLoS One. 2013 Jan 9;8(1):e53303. doi: 10.1371/journal.pone.0053303 (PMC3541250; doi:10.1371/journal.pone.0053303)
Supplement: Table S1 — Variation amplitude and time-average difference in depth-averaged Total Alkalinity (TA) between reef waters and offshore waters over a 24-hour period. (DOC) [file pone.0053303.s010.doc]

## Table S1

Variation amplitude $\left(A_{\text{TA}}\right)$ and time-average difference $\left(\delta_{\text{TA}}\right)$ in depth-averaged Total Alkalinity (TA in μeq kg^-1^) between reef waters and offshore waters over a 24-hour period. All data shown represent values averaged across the line transects and lagoon area identified in Fig. 2B.

|  | **forereef** | |  | **backreef** | |  | **lagoon** | |
| --- | --- | --- | --- | --- | --- | --- | --- | --- |
| **Simulation** | $A_{\text{TA}}$ | $\delta_{\text{TA}}$ |  | $A_{\text{TA}}$ | $\delta_{\text{TA}}$ |  | $A_{\text{TA}}$ | $\delta_{\text{TA}}$ |
| Central Case | 5 | -13 |  | 14 | -26 |  | 15 | -26 |
| *H*_0_ = 0.5 m | 6 | -10 |  | 43 | -40 |  | 40 | -40 |
| *H*_0_ = 1 m | 6 | -12 |  | 20 | -32 |  | 21 | -31 |
| *H*_0_ = 2 m | 5 | -12 |  | 12 | -21 |  | 13 | -21 |
| *H*_0_ = 3 m | 4 | -8 |  | 10 | -15 |  | 11 | -15 |
| *h*_r_ = 0.5 m | 4 | -11 |  | 38 | -38 |  | 34 | -39 |
| *h*_r_ = 0.7 m | 5 | -13 |  | 23 | -32 |  | 23 | -32 |
| *h*_r_ = 1.5 m | 6 | -13 |  | 11 | -23 |  | 12 | -22 |
| *h*_r_ = 2.0 m | 6 | -13 |  | 10 | -22 |  | 11 | -21 |
| *L*_r_ = 250 m | 4 | -10 |  | 7 | -17 |  | 8 | -17 |
| *L*_r_ = 500 m | 7 | -17 |  | 24 | -37 |  | 24 | -36 |
| *L*_r_ = 1000 m | 6 | -19 |  | 36 | -46 |  | 35 | -45 |
| *h*_c_ = 3 m | 8 | -18 |  | 22 | -39 |  | 23 | -39 |
| *h*_c_ = 4.5 m | 7 | -15 |  | 16 | -31 |  | 17 | -30 |
| *h*_c_ = 10 m | 3 | -8 |  | 14 | -19 |  | 14 | -19 |
| *W*_c_ = 200 m | 6 | -16 |  | 20 | -33 |  | 21 | -32 |
| *W*_c_ = 300 m | 6 | -15 |  | 17 | -30 |  | 18 | -29 |
| *W*_c_ = 450 m | 6 | -14 |  | 15 | -28 |  | 16 | -27 |
| *W*_c_ = 1200 m | 4 | -10 |  | 14 | -22 |  | 15 | -23 |
| *η*_sea_ = +0.5 m | 5 | -12 |  | 9 | -21 |  | 10 | -20 |
| *η*_sea_ = +1 m | 5 | -11 |  | 8 | -18 |  | 9 | -17 |
| *η*_sea_ = +2 m | 4 | -8 |  | 7 | -15 |  | 8 | -14 |
| *η*_sea_ = +4 m | 3 | -2 |  | 8 | -8 |  | 5 | -8 |
| *P* = 150 | 2 | -4 |  | 3 | -7 |  | 4 | -8 |
| *P* = 330 | 3 | -7 |  | 7 | -14 |  | 8 | -14 |
| *P* = 1000 | 8 | -20 |  | 22 | -40 |  | 23 | -39 |
| *P* = 1500 | 12 | -29 |  | 34 | -59 |  | 35 | -57 |
| *G*_net_*:P = 0%* | 0 | 0 |  | 0 | 0 |  | 0 | 0 |
| *G*_net_*:P = 40%* | 11 | -26 |  | 29 | -53 |  | 30 | -52 |
| *P*_lag_ = 330 | 9 | -20 |  | 14 | -35 |  | 16 | -37 |
| *P*_lag_ = 330, *h*_c_ = 3 m | 12 | -26 |  | 21 | -49 |  | 25 | -54 |
| *U*_off_ = 0.125 m s^-1^ | 6 | -15 |  | 16 | -26 |  | 17 | -25 |
| *L*_lag_ = 1500 m, *h*_c_ = 20 m | 2 | -1 |  | 17 | -10 |  | 10 | -9 |
