# Supplementary material for: Physical and Biological Controls on the Carbonate Chemistry of Coral Reef Waters: Effects of Metabolism, Wave Forcing, Sea Level, and Geomorphology
Source: PLoS One. 2013 Jan 9;8(1):e53303. doi: 10.1371/journal.pone.0053303 (PMC3541250; doi:10.1371/journal.pone.0053303)
Supplement: Table S2 — Variation amplitude and time-average difference in depth-averaged Dissolved Inorganic Carbon (DIC) between reef waters and offshore waters over a 24-hour period. (DOC) [file pone.0053303.s011.doc]

## Table S2

Variation amplitude $\left(A_{\text{DIC}}\right)$ and time-average difference $\left(\delta_{\text{DIC}}\right)$ in depth-averaged Dissolved Inorganic Carbon (DIC in μmol kg^-1^) between reef waters and offshore waters over a 24-hour period. All data shown represent values averaged across the line transects and lagoon area identified in Fig. 2B.

|  | **forereef** | |  | **backreef** | |  | **lagoon** | |  |
| --- | --- | --- | --- | --- | --- | --- | --- | --- | --- |
| **Simulation** | $A_{\text{DIC}}$ | $\delta_{\text{DIC}}$ |  | $A_{\text{DIC}}$ | $\delta_{\text{DIC}}$ |  | $A_{\text{DIC}}$ | $\delta_{\text{DIC}}$ |  |
| Central Case | 13 | -12 |  | 40 | -23 |  | 40 | -23 |  |
| *H*_0_ = 0.5 m | 18 | -7 |  | 120 | -32 |  | 109 | -37 |  |
| *H*_0_ = 1 m | 13 | -11 |  | 57 | -28 |  | 56 | -28 |  |
| *H*_0_ = 2 m | 13 | -10 |  | 34 | -18 |  | 34 | -18 |  |
| *H*_0_ = 3 m | 12 | -7 |  | 29 | -12 |  | 29 | -12 |  |
| *h*_r_ = 0.5 m | 9 | -9 |  | 107 | -31 |  | 93 | -35 |  |
| *h*_r_ = 0.7 m | 11 | -12 |  | 64 | -27 |  | 62 | -29 |  |
| *h*_r_ = 1.5 m | 14 | -12 |  | 30 | -20 |  | 31 | -20 |  |
| *h*_r_ = 2.0 m | 13 | -12 |  | 27 | -19 |  | 28 | -18 |  |
| *L*_r_ = 250 m | 10 | -9 |  | 21 | -15 |  | 22 | -15 |  |
| *L*_r_ = 500 m | 15 | -15 |  | 68 | -32 |  | 66 | -32 |  |
| *L*_r_ = 1000 m | 13 | -17 |  | 102 | -39 |  | 95 | -41 |  |
| *h*_c_ = 3 m | 21 | -16 |  | 61 | -32 |  | 62 | -33 |  |
| *h*_c_ = 4.5 m | 17 | -14 |  | 45 | -26 |  | 45 | -26 |  |
| *h*_c_ = 10 m | 8 | -7 |  | 40 | -16 |  | 37 | -17 |  |
| *W*_c_ = 200 m | 14 | -15 |  | 57 | -28 |  | 55 | -28 |  |
| *W*_c_ = 300 m | 14 | -14 |  | 48 | -26 |  | 47 | -26 |  |
| *W*_c_ = 450 m | 14 | -13 |  | 42 | -24 |  | 42 | -24 |  |
| *W*_c_ = 1200 m | 11 | -8 |  | 39 | -19 |  | 39 | -20 |  |
| *η*_sea_ = +0.5 m | 13 | -11 |  | 27 | -18 |  | 27 | -18 |  |
| *η*_sea_ = +1 m | 12 | -10 |  | 22 | -16 |  | 22 | -15 |  |
| *η*_sea_ = +2 m | 11 | -8 |  | 19 | -13 |  | 19 | -12 |  |
| *η*_sea_ = +4 m | 9 | -2 |  | 22 | -6 |  | 14 | -6 |  |
| *P* = 150 | 4 | -4 |  | 9 | -6 |  | 9 | -7 |  |
| *P* = 330 | 7 | -7 |  | 20 | -12 |  | 20 | -13 |  |
| *P* = 1000 | 19 | -18 |  | 62 | -34 |  | 61 | -34 |  |
| *P* = 1500 | 28 | -26 |  | 93 | -51 |  | 92 | -50 |  |
| *G*_net_*:P = 0%* | 11 | -5 |  | 34 | -10 |  | 33 | -10 |  |
| *G*_net_*:P = 40%* | 16 | -19 |  | 47 | -36 |  | 48 | -36 |  |
| *P*_lag_ = 330 | 22 | -18 |  | 38 | -31 |  | 42 | -33 |  |
| *P*_lag_ = 330, *h*_c_ = 3 m | 33 | -23 |  | 57 | -42 |  | 67 | -47 |  |
| *U*_off_ = 0.125 m s^-1^ | 14 | -17 |  | 44 | -25 |  | 45 | -25 |  |
| *L*_lag_ = 1500 m, *h*_c_ = 20 m | 5 | -4 |  | 48 | -10 |  | 28 | -10 |  |
