# Supplementary material for: Physical and Biological Controls on the Carbonate Chemistry of Coral Reef Waters: Effects of Metabolism, Wave Forcing, Sea Level, and Geomorphology
Source: PLoS One. 2013 Jan 9;8(1):e53303. doi: 10.1371/journal.pone.0053303 (PMC3541250; doi:10.1371/journal.pone.0053303)
Supplement: Table S3 — Variation amplitude and time-average difference in depth-averaged p CO2 between reef waters and offshore waters over a 24-hour period. (DOC) [file pone.0053303.s012.doc]

## Table S3

Variation amplitude $\left(A_{\text{pCO}_{2}}\right)$ and time-average difference $\left(\delta_{\text{pCO}_{2}}\right)$ in depth-averaged *p*CO_2_ in μatm between reef waters and offshore waters over a 24-hour period. All data shown represent values averaged across the line transects and lagoon area identified in Fig. 2B.

|  | **forereef** | |  | **backreef** | |  | **lagoon** | |  |
| --- | --- | --- | --- | --- | --- | --- | --- | --- | --- |
| **Simulation** | $A_{\text{pCO}_{2}}$ | $\delta_{\text{pCO}_{2}}$ |  | $A_{\text{pCO}_{2}}$ | $\delta_{\text{pCO}_{2}}$ |  | $A_{\text{pCO}_{2}}$ | $\delta_{\text{pCO}_{2}}$ |  |
| Central Case | 16 | -2 |  | 52 | -1 |  | 50 | -2 |  |
| *H*_0_ = 0.5 m | 25 | 2 |  | 152 | 20 |  | 135 | 7 |  |
| *H*_0_ = 1 m | 16 | -2 |  | 72 | 1 |  | 69 | -2 |  |
| *H*_0_ = 2 m | 17 | -1 |  | 45 | 1 |  | 44 | 0 |  |
| *H*_0_ = 3 m | 16 | 0 |  | 38 | 2 |  | 38 | 2 |  |
| *h*_r_ = 0.5 m | 12 | -1 |  | 132 | 14 |  | 114 | 6 |  |
| *h*_r_ = 0.7 m | 14 | -2 |  | 81 | 3 |  | 76 | 0 |  |
| *h*_r_ = 1.5 m | 17 | -2 |  | 40 | -1 |  | 39 | -2 |  |
| *h*_r_ = 2.0 m | 17 | -2 |  | 36 | -1 |  | 35 | -2 |  |
| *L*_r_ = 250 m | 13 | -2 |  | 29 | -1 |  | 29 | -2 |  |
| *L*_r_ = 500 m | 18 | -3 |  | 85 | 3 |  | 80 | 0 |  |
| *L*_r_ = 1000 m | 16 | -3 |  | 127 | 10 |  | 116 | 4 |  |
| *h*_c_ = 3 m | 27 | -2 |  | 79 | 3 |  | 76 | 1 |  |
| *h*_c_ = 4.5 m | 21 | -2 |  | 58 | 0 |  | 57 | -1 |  |
| *h*_c_ = 10 m | 10 | -1 |  | 50 | 1 |  | 47 | -1 |  |
| *W*_c_ = 200 m | 17 | -3 |  | 73 | 1 |  | 69 | -1 |  |
| *W*_c_ = 300 m | 17 | -3 |  | 62 | 0 |  | 59 | -2 |  |
| *W*_c_ = 450 m | 17 | -3 |  | 55 | -1 |  | 53 | -2 |  |
| *W*_c_ = 1200 m | 14 | -1 |  | 50 | 0 |  | 49 | -1 |  |
| *η*_sea_ = +0.5 m | 17 | -2 |  | 35 | -2 |  | 35 | -2 |  |
| *η*_sea_ = +1 m | 15 | -2 |  | 29 | -2 |  | 29 | -2 |  |
| *η*_sea_ = +2 m | 13 | -2 |  | 25 | -1 |  | 24 | -1 |  |
| *η*_sea_ = +4 m | 12 | 0 |  | 28 | 2 |  | 18 | 1 |  |
| *P* = 150 | 5 | -1 |  | 11 | -1 |  | 12 | -1 |  |
| *P* = 330 | 9 | -1 |  | 26 | -1 |  | 26 | -1 |  |
| *P* = 1000 | 24 | -3 |  | 79 | 1 |  | 75 | 0 |  |
| *P* = 1500 | 35 | -4 |  | 118 | 6 |  | 111 | 3 |  |
| *G*_net_*:P = 0%* | 19 | -9 |  | 56 | -14 |  | 55 | -15 |  |
| *G*_net_*:P = 40%* | 14 | 5 |  | 48 | 15 |  | 46 | 13 |  |
| *P*_lag_ = 330 | 28 | -3 |  | 50 | -2 |  | 53 | -3 |  |
| *P*_lag_ = 330, *h*_c_ = 3 m | 42 | -1 |  | 75 | 1 |  | 85 | 1 |  |
| *U*_off_ = 0.125 m s^-1^ | 17 | -8 |  | 57 | -5 |  | 56 | -6 |  |
| *L*_lag_ = 1500 m, *h*_c_ = 20 m | 7 | -5 |  | 61 | -1 |  | 36 | -5 |  |
