# Supplementary material for: Physical and Biological Controls on the Carbonate Chemistry of Coral Reef Waters: Effects of Metabolism, Wave Forcing, Sea Level, and Geomorphology
Source: PLoS One. 2013 Jan 9;8(1):e53303. doi: 10.1371/journal.pone.0053303 (PMC3541250; doi:10.1371/journal.pone.0053303)
Supplement: Table S4 — Variation amplitude and time-average difference in depth-averaged pH between reef waters and offshore waters over a 24-hour period. (DOC) [file pone.0053303.s013.doc]

## Table S4

Variation amplitude $\left(A_{\text{pH}}\right)$ and time-average difference $\left(\delta_{\text{pH}}\right)$ in depth-averaged pH (as defined on the total scale) between reef waters and offshore waters over a 24-hour period. All data shown represent values averaged across the line transects and lagoon area identified in Fig. 2B.

|  | **forereef** | |  | **backreef** | |  | **lagoon** | |  |
| --- | --- | --- | --- | --- | --- | --- | --- | --- | --- |
| **Simulation** | $A_{\text{pH}}$ | $\delta_{\text{pH}}$ |  | $A_{\text{pH}}$ | $\delta_{\text{pH}}$ |  | $A_{\text{pH}}$ | $\delta_{\text{pH}}$ |  |
| Central Case | 0.01 | 0.00 |  | 0.05 | 0.00 |  | 0.05 | 0.00 |  |
| *H*_0_ = 0.5 m | 0.02 | 0.00 |  | 0.14 | -0.01 |  | 0.12 | 0.00 |  |
| *H*_0_ = 1 m | 0.01 | 0.00 |  | 0.07 | 0.00 |  | 0.06 | 0.00 |  |
| *H*_0_ = 2 m | 0.01 | 0.00 |  | 0.04 | 0.00 |  | 0.04 | 0.00 |  |
| *H*_0_ = 3 m | 0.01 | 0.00 |  | 0.03 | 0.00 |  | 0.03 | 0.00 |  |
| *h*_r_ = 0.5 m | 0.01 | 0.00 |  | 0.12 | -0.01 |  | 0.11 | 0.00 |  |
| *h*_r_ = 0.7 m | 0.01 | 0.00 |  | 0.07 | 0.00 |  | 0.07 | 0.00 |  |
| *h*_r_ = 1.5 m | 0.02 | 0.00 |  | 0.04 | 0.00 |  | 0.03 | 0.00 |  |
| *h*_r_ = 2.0 m | 0.01 | 0.00 |  | 0.03 | 0.00 |  | 0.03 | 0.00 |  |
| *L*_r_ = 250 m | 0.01 | 0.00 |  | 0.03 | 0.00 |  | 0.03 | 0.00 |  |
| *L*_r_ = 500 m | 0.02 | 0.00 |  | 0.08 | 0.00 |  | 0.07 | 0.00 |  |
| *L*_r_ = 1000 m | 0.01 | 0.00 |  | 0.12 | 0.00 |  | 0.11 | 0.00 |  |
| *h*_c_ = 3 m | 0.02 | 0.00 |  | 0.07 | 0.00 |  | 0.07 | 0.00 |  |
| *h*_c_ = 4.5 m | 0.02 | 0.00 |  | 0.05 | 0.00 |  | 0.05 | 0.00 |  |
| *h*_c_ = 10 m | 0.01 | 0.00 |  | 0.05 | 0.00 |  | 0.04 | 0.00 |  |
| *W*_c_ = 200 m | 0.01 | 0.00 |  | 0.07 | 0.00 |  | 0.06 | 0.00 |  |
| *W*_c_ = 300 m | 0.02 | 0.00 |  | 0.06 | 0.00 |  | 0.05 | 0.00 |  |
| *W*_c_ = 450 m | 0.01 | 0.00 |  | 0.05 | 0.00 |  | 0.05 | 0.00 |  |
| *W*_c_ = 1200 m | 0.01 | 0.00 |  | 0.05 | 0.00 |  | 0.04 | 0.00 |  |
| *η*_sea_ = +0.5 m | 0.01 | 0.00 |  | 0.03 | 0.00 |  | 0.03 | 0.00 |  |
| *η*_sea_ = +1 m | 0.01 | 0.00 |  | 0.03 | 0.00 |  | 0.03 | 0.00 |  |
| *η*_sea_ = +2 m | 0.01 | 0.00 |  | 0.02 | 0.00 |  | 0.02 | 0.00 |  |
| *η*_sea_ = +4 m | 0.01 | 0.00 |  | 0.02 | 0.00 |  | 0.02 | 0.00 |  |
| *P* = 150 | 0.00 | 0.00 |  | 0.01 | 0.00 |  | 0.01 | 0.00 |  |
| *P* = 330 | 0.01 | 0.00 |  | 0.02 | 0.00 |  | 0.02 | 0.00 |  |
| *P* = 1000 | 0.02 | 0.00 |  | 0.07 | 0.00 |  | 0.07 | 0.00 |  |
| *P* = 1500 | 0.03 | 0.00 |  | 0.11 | 0.00 |  | 0.10 | 0.00 |  |
| *G*_net_*:P = 0%* | 0.02 | 0.01 |  | 0.05 | 0.02 |  | 0.05 | 0.02 |  |
| *G*_net_*:P = 40%* | 0.01 | 0.00 |  | 0.04 | -0.02 |  | 0.04 | -0.01 |  |
| *P*_lag_ = 330 | 0.02 | 0.00 |  | 0.04 | 0.00 |  | 0.05 | 0.00 |  |
| *P*_lag_ = 330, *h*_c_ = 3 m | 0.04 | 0.00 |  | 0.07 | 0.00 |  | 0.08 | 0.00 |  |
| *U*_off_ = 0.125 m s^-1^ | 0.01 | 0.01 |  | 0.05 | 0.00 |  | 0.05 | 0.00 |  |
| *L*_lag_ = 1500 m, *h*_c_ = 20 m | 0.01 | 0.00 |  | 0.05 | 0.00 |  | 0.03 | 0.00 |  |
